# NavegApp, a serious game for assessing spatial cognition: Diagnostic accuracy in preclinical and prodromal Alzheimer’s disease

**DOI:** 10.1371/journal.pdig.0001521

**Published:** 2026-07-10

**Authors:** Juan Pablo Sánchez-Escudero, Diego Camilo Díaz González, David Fernando Aguillón-Niño, Mauricio A. Garcia-Barrera, Daniel Camilo Aguirre-Acevedo, Natalia Trujillo-Orrego

**Affiliations:** 1 Group of Epidemiology, University of Antioquia, Medellín, Colombia; 2 Faculty of Social, Health and Well-being Sciences, Luis Amigó Catholic University, Apartadó, Colombia; 3 Group of Neurosciences of Antioquia, University of Antioquia, Medellín, Colombia; 4 Department of Psychology & Institute on Aging and Lifelong Health, University of Victoria, Victoria, British Columbia, Canada; 5 School of Medicine, University of Antioquia, Medellín, Colombia; 6 Mental Health Research Group, Universidad de Antioquia, Medellín, Colombia; 7 Atlantic Fellowship in Equity in Brain Health, Global Brain Health Institute, University of California, San Francisco, California, United States of America; 8 Stempel College of Public Health and Social Work, Florida International University, Miami, Florida, United States of America; The Hong Kong Polytechnic University, HONG KONG

## Abstract

Alzheimer’s Disease (AD) is the leading cause of dementia worldwide, yet early detection remains challenging due to limited access to biomarker-based diagnostic tools, especially in low- and middle-income countries. This study aims to evaluate the diagnostic accuracy of NavegApp, a serious game developed to assess Spatial Cognition (SC), in distinguishing individuals at various stages of AD, including asymptomatic and symptomatic PSEN1-E280A mutation carriers. A cross-sectional sample of 226 participants underwent neurological and neuropsychological evaluations alongside NavegApp targeting allocentric navigation, mental rotation, and visuospatial memory. Results showed excellent diagnostic accuracy for distinguishing symptomatic PSEN1-E280A carriers from asymptomatic carriers and healthy controls, particularly in allocentric navigation metrics (AUC-ROC = 0.94–0.97). However, in asymptomatic participants, diagnostic performance was modest (AUC ≈ 0.57–0.60), indicating limited discriminative capacity at the preclinical stage. Cross-sectional comparisons detected prodromal-stage deficits in visuospatial and mental rotation tasks, whereas diagnostic accuracy for distinguishing sporadic MCI from health controls was moderate to low. These findings demonstrate the feasibility of NavegApp as a digital tool for cognitive assessment, with potential applicability in cognitive screening for underserved communities. Further research must validate its use across diverse settings and establish its integration into clinical practice for early AD detection.

## Author summary

Alzheimer’s disease is a growing global concern, and early detection is essential to help people receive timely care. However, many current diagnostic tools rely on expensive technologies that are often unavailable in low-resource settings. In this study, we tested NavegApp, a serious game we developed to evaluate people’s spatial cognition abilities, which may be affected in the early stages of Alzheimer’s disease. We assessed 226 individuals, including those with a genetic risk for early-onset Alzheimer’s, those with mild cognitive symptoms, and healthy individuals. Our results show that NavegApp can accurately detect cognitive impairments in individuals with early symptoms, especially those affecting navigation and visuospatial skills. Although the tool was less effective at identifying very early (preclinical) changes, it performed well in detecting prodromal Alzheimer’s, when symptoms begin to emerge. Because NavegApp is low-cost and easy to use on a tablet, it has potential to bring cognitive assessment to underserved communities after validation in diverse populations, training of local health workers, and demonstration of clinical utility. We believe this tool has the potential to improve access to early detection, supporting interventions and research efforts in areas where traditional diagnostic methods are not available.

## Introduction

Alzheimer’s Disease (AD) is the leading cause of dementia worldwide, accounting for approximately 70% of cases [1,2]. With an aging global population, the prevalence and incidence of AD are projected to increase, affecting around 152 million people by 2050 [3,4]. Beyond the profound social and emotional load to patients and their families, AD imposes considerable economic burdens, with healthcare costs reaching approximately $277 billion and contributing to the loss of nearly 33.1 million disability-adjusted life-years, positioning it as a critical threat to health and social systems worldwide [4,5]. The absence of effective, widely available pharmacological treatments underscores early detection’s importance in facilitating preventive interventions. Early diagnosis can maximize the potential of emerging treatments, delay symptom onset, and provide additional functional years to individuals at risk [1,3].

Preclinical detection of Alzheimer’s disease primarily relies on identifying molecular signatures of the disease before any cognitive impairment can be detected [6]. Biomarkers in cerebrospinal fluid and neuroimaging have proven accurate for detecting AD years before cognitive or functional impairments manifest [7]. However, these techniques are costly and largely inaccessible, particularly in underserved or remote settings [6]. As a result, accurate early detection of AD risk remains limited in low- and middle-income countries and in populations with restricted access to healthcare and education systems. Consequently, exploring cognitive markers associated with AD has emerged as a potential solution for early detection [8]. In contrast to biomarkers, cognitive markers can be obtained non-invasively through neuropsychological assessments, providing valuable evidence on the functional and cognitive decline biomarkers are intended to detect [8].

Episodic memory has traditionally been the cornerstone of cognitive assessment in AD [9]. However, as deficits in this cognitive process are not exclusive to AD, they may lead to underestimations of cognitive decline and reduced sensitivity in early detection [9]. Models of disease progression, such as the Braak model, suggest that deficits in episodic memory may be preceded by subtle impairments in other cognitive processes linked to sub-hippocampal structures [10]. This insight has shifted research focus toward identifying alternative cognitive markers that align with the temporal and spatial progression of AD throughout the brain [11].

Accrued evidence has highlighted the potential of Spatial Cognition (SC) measures to distinguish between normal and pathological aging associated with AD both in preclinical and prodromic stages [9,12–16]. SC involves mental representations of spatial relationships in the environment and is mediated by brain structures within the medial temporal and parietal lobes, particularly in the right hemisphere [17]. Given the involvement of multiple brain regions in spatial processing, neurodegenerative diseases like AD impact SC in specific ways as the disease progresses [17]. Thus, distinct SC deficits have been associated with neurodegeneration in sub-hippocampal areas, such as the entorhinal cortex in preclinical AD stages, and parietal structures, such as the precuneus, during the prodromal stage. These deficits lead to impairments in cognitive processes, including allocentric navigation, mental rotation, and visuospatial memory [9,16]. Although SC holds promise as a source of cognitive markers for early AD detection, there remains no standardized protocol for its assessment in clinical settings [17].

Most existing tasks and tests for measuring SC components in the context of early AD detection focus primarily on Virtual Reality (VR)-based spatial navigation performance [18]. While valuable, VR tasks require substantial physical space and expensive equipment, which limits its implementation out of research settings [19,20]. Additionally, many of these tasks lack validation on critical metrics such as content validity, usability and digital ergonomics, which limits their suitability for clinical application, especially in low- and middle-income countries [18,21,22]. Other limitations of these tools include language barriers and restricted software access, which hinder the ability to test specific hypotheses in research environments such as item adequacy, changes in instructions or customized tutorials for people with low education or technological literacy, slowing down their validation and adaptation for diverse populations.

In response to these limitations, innovative digital technologies, such as Serious Games (SG) for cognitive assessment, have gained traction in research settings in recent years [18]. By leveraging advancements in video game engines and high-level language programming, researchers across disciplines are increasingly adapting and developing tasks to assess complex behavioural outcomes, including SC [23]. An example is NavegApp, a serious game-based platform explicitly designed to assess SC-related cognitive processes [23]. This tool comprises three tasks that may be sensitive to early AD neuropathology. Additionally, NavegApp offers features such as automatic data collection, easy administration, and low cost, thus overcoming the primary limitations associated with traditional paper-and-pencil neuropsychological assessments and VR-based tasks [8,24].

Customized SGs, such as NavegApp, adapted for underprivileged communities, offer a valuable opportunity to gather critical information on cognitive changes across the AD spectrum in populations at heightened risk due to socioeconomic disparity, social health determinants, and genetic predispositions [25]. One such group are the carriers of the PSEN1-E280A genetic variant, a Colombian cohort that represents the world’s largest kindred with a causative mutation for early-onset familial AD [26]. Individuals with the PSEN1-E280A mutation typically report memory complaints without significant lifestyle impact by age 38, progress to Mild Cognitive Impairment (MCI) around age 44, and develop dementia by approximately age 59 [27]. Due to similarities in pathophysiology and progression between PSEN1-E280A carriers and sporadic AD cases, studying this cohort provides valuable insights for identifying cognitive markers of AD.

While NavegApp has proven high usability, digital ergonomics and content validity [23], further evidence is needed to establish its diagnostic accuracy. Thus, this study aims to evaluate NavegApp’s ability to distinguish individuals at different stages of AD. To this end, we assessed participants with both genetic and sporadic forms of AD, comparing the performance of asymptomatic PSEN1-E280A carriers, asymptomatic PSEN1-E280A non-carriers and symptomatic individuals carrying the mutation. In addition, a group of individuals with sporadic MCI were compared with age-matched controls.

Based on previous literature [9–11,18,28], we formulated two primary hypotheses. First, allocentric navigation deficits would be detectable in asymptomatic individuals, with minimal or no impairment in mental rotation or visuospatial short-term memory. Second, individuals with sporadic MCI would exhibit deficits in allocentric navigation, mental rotation, and visuospatial short-term memory compared to healthy counterparts. Evidence from NavegApp’s diagnostic accuracy could shed light on its potential as a viable digital cognitive assessment tool for underserved populations with currently limited access to cognitive screening initiatives. We anticipate that insights gained from the application of NavegApp to PSEN1-E280A carriers will be valuable for informing and supporting cognitive monitoring programs, customizing treatments, and contributing to population-level initiatives.

## Materials and methods

### Study design

A cross-sectional study was conducted from September 2023 to July 2024. Participants were recruited using non-probabilistic convenience sampling through telephone contact and in-person invitations. Most were identified from institutional research databases of the Group of Neurosciences of Antioquia, where they had previously consented to future contact. Additional asymptomatic participants were recruited through family referrals.

All participants were contacted directly and invited to attend an in-person clinical evaluation in Medellín, Antioquia, Colombia. Eligibility criteria were verified during standardized neurological and neuropsychological assessments conducted in outpatient clinical settings by trained physicians and neuropsychologists. Basic literacy skills and the absence of perceptual or motor deficits interfering with assessment were confirmed during the clinical evaluation. All individuals who attended the scheduled evaluation were screened for eligibility. Participants received monetary compensation equivalent to one day of the legally established minimum wage in Colombia for the corresponding year.

### Population and sample

**Fig 1:**
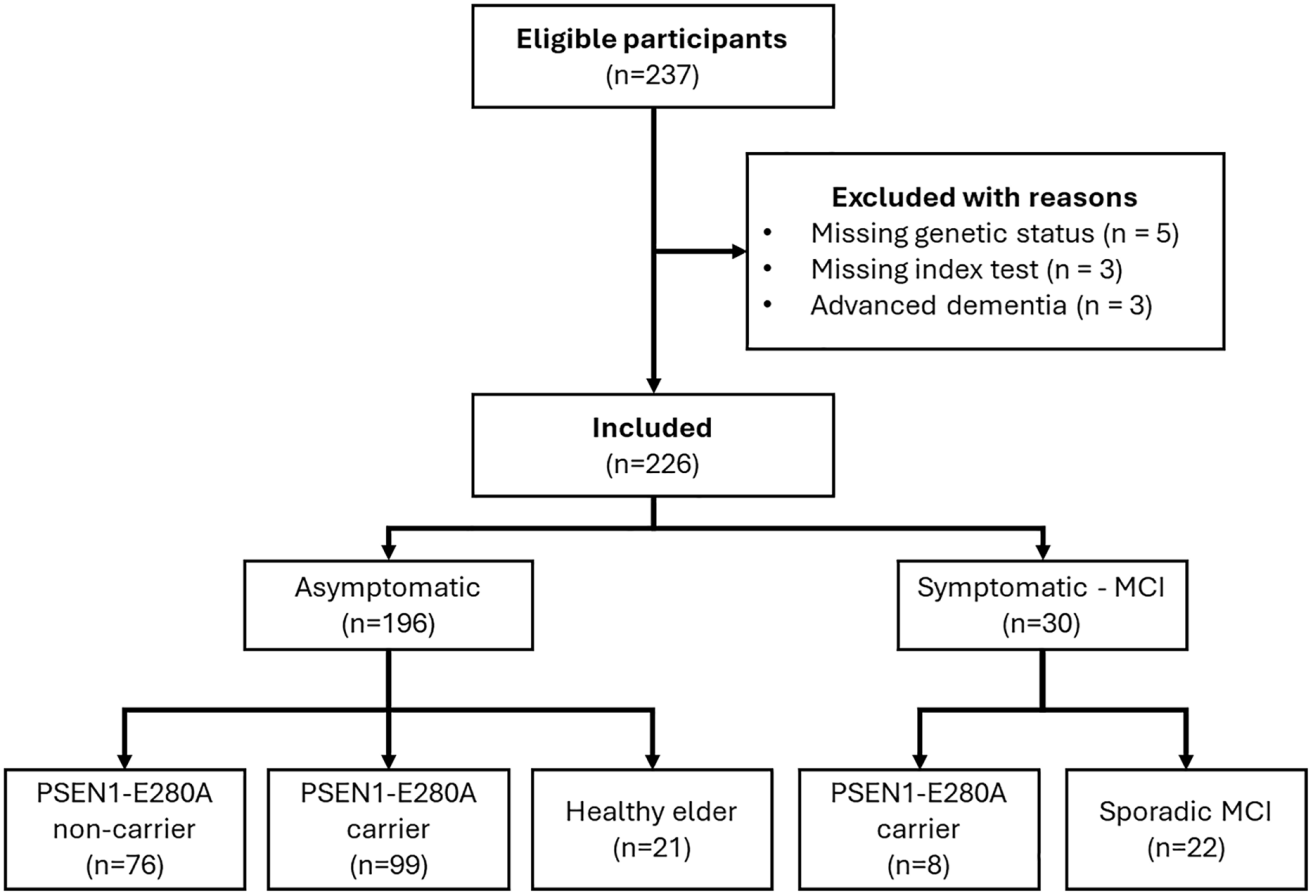
Flowchart of participants included in the study.

A total of 237 participants were initially recruited from existing databases and multiple sources. Of these, 11 were excluded based on the exclusion criteria outlined in Fig 1. This resulted in a final sample of 226 participants (63.7% female) included in the analyses. Inclusion criteria required participants to be adults without a prior diagnosis of dementia or other neurodegenerative disorders, possessing basic literacy skills, and free from perceptual or motor deficits that could interfere with clinical assessments. Participants were then classified into one of five groups according to their neurological clinical evaluation and genetic profile.

The criteria established by the International Working Group on Mild Cognitive Impairment, grounded in the classical Petersen criteria [29], were used to determine the cognitive status of participants. Thus, asymptomatic participants were defined as individuals without subjective cognitive complaints, showing no cognitive decline and no significant deficits in non-memory cognitive domains, as stated by absolute Z-scores >2 in at least two cognitive domains different from memory. These participants were further stratified into PSEN1-E280A carriers and non-carriers based on genetic assessments. Symptomatic participants were similarly classified according to clinical neurological and neuropsychological evaluations, with MCI subgroups identified as either PSEN1-E280A (MCI-C) carriers or individuals with sporadic MCI (sMCI), depending on their genetic status. Cognitively unimpaired participants were stratified as PSEN1-E280A carriers (CU-C) and non-carriers (CU-NC). Additionally, a group of cognitively healthy PSEN1-E280A non-carriers aged >65 years (HE-NC) was included for comparison.

### Ethical considerations

All the research procedures were performed following Colombian regulations for research involving human beings [30]. The study’s objectives were explained beforehand, and all the participants were informed and accepted their participation by accepting the informed consent form. All participants provided informed consent, which was approved by the Ethics Committee of the School of Public Health (Act 21030002-00153-2022).

Neurological and neuropsychological assessments were carried out by trained personnel with extensive experience assessing individuals from this cohort. Examiners were blinded to the genetic status of participants to avoid diagnostic and clinical review bias. All the participants were examined using the same protocol version and tested to avoid work-up bias. The scores of the index test did not influence the neurological or neuropsychological assessment, avoiding, therefore, incorporation bias. All test results were double-checked and uploaded to the medical registry system in conformity with the good practices for clinical data management. For individuals suspected of MCI, relatives or caregivers were present during the consent process to ensure clear communication about the study. Their understanding of the study’s details was verified, and they provided permission for those under their care to participate.

#### Procedure.

Research team members contacted eligible individuals and invited them to participate. Once consent was granted, participants underwent neurological and neuropsychological examinations. All participants who provided informed consent completed the full evaluation protocol, and no dropouts occurred during the study.

The index test, NavegApp, was administered in a clinical setting under standardized conditions using tablet devices, after completion of the neurological and neuropsychological assessments, within the same outpatient facilities. NavegApp was run on Lenovo M10 FHD Plus tablets equipped with a 10.3-inch touchscreen and operating on Android 10. All devices were configured uniformly across participants, and no modifications to default task parameters were implemented during data collection, ensuring consistency in task presentation and data acquisition. All participants were explained how to use a tablet and NavegApp according to the instructions in the app’s manual and previous training. Physicians solved all the questions that emerged during the SG application.

### Test methods

#### Neurological assessment.

The neurological assessment consisted of a general medical examination in which information was collected on medical, neurological and family history, risk factors and medical history, and functional scales to support the diagnosis. Biological sex assigned at birth was assessed during the medical examination and confirmed by participant self-report. Diagnostic status was determined from the joint analysis of the evidence provided by the medical and neurological assessment. All medical, neurological and index test (i.e., NavegApp) examinations were performed on the same day, hours apart. A clinical staff composed of physicians and neuropsychologists with expertise in the diagnosis of dementia established the diagnosis in cases with equivocal test results.

#### Neuropsychological assessment.

All the participants underwent neuropsychological examination using a comprehensive neuropsychological test battery adapted for the Spanish-speaking Colombian population [31], which includes the CERAD-Col battery [32,33], Boston Naming Test (BNT-15), Rey-Osterrieth Complex Figure (RCFT), Raven’s Progressive Matrices, Trail Making Test-A (TMT-A), WAIS-III Digit Symbol, Phonemic Fluency (FAS) and the Wisconsin Card Sorting Task (WCST) [31]. The neurological assessment was performed in person and did not rely on medical records. Functional status was assessed using standardized instruments, including the Barthel Index for basic activities of daily living, the Lawton and Brody scale for instrumental activities of daily living, and the Katz Index of Independence in Activities of Daily Living. All assessments followed validated protocols routinely applied in this population [31].

#### Genetic assessment.

Genomic DNA was extracted from blood samples using standard protocols for genetic analysis, and PSEN1-E280A characterization was conducted following previously established methods [34]. The genomic DNA was amplified using the primers PS1-S (5´ AACAGCTCAGGAGAGGAATG 3´) and PS1-AS (5´ GATGAGACAAGTNCCNTGAA 3´). The PSEN1-E280A mutation and the intronic polymorphism were detected through PCR with mismatch primers, followed by enzymatic digestion of the PCR products with Bsm1 and BamH1.

#### Index test: NavegApp.

NavegApp is an SG-based platform developed to assess three main components of SC, described in previous literature as potentially sensitive to AD [12,35,36]. The application was developed using the Unity game engine (version 2022.1.20f1). The beta version of NavegApp was used for all assessments; details of its development, content validity, usability, and digital ergonomics have been described elsewhere [23].

NavegApp encompasses three gamified tasks adapted from established protocols in the literature and tailored for use in people with neurodegenerative diseases [23]. The gamified Hidden Goal Task (gHGT) assesses allocentric navigation in a 2D environment based on the use of distal cues, capturing three primary metrics: mean error to goal (distance to the hidden target), path length, and path time [15,37]. Path length represents the mean Euclidean distance traversed across trials, whereas path time reflects the mean trial completion time in milliseconds.

The gamified Corsi Block Tapping Test (gCorsi) assesses short-term visuospatial memory through touchscreen interactions, capturing performance metrics such as span and average reaction time for the forward and backward conditions [38,39]. Finally, the gamified Mental Rotation Task (gMRT) examines participants’ ability to mentally rotate 2D objects across three conditions (i.e., 0°, 90°, 180°) and select them from a set of stimuli disposed on the screen [12]. Further details on the content validity, usability, and digital ergonomics of NavegApp are provided elsewhere [23].

#### Statistical analysis.

Statistical analysis was performed using R Statistical Software V 4.4.1 [40] and RStudio V2024.09.0 [41]. Missing data on the index test metrics were detected, quantified, and treated in accordance with the recommendations for missing data management [42,43]. Outliers were detected through visual inspection and addressed using the winsorization method [44,45]. Additional results describing the distribution of NavegApp measures before and after missing-data imputation are provided in S1 Fig. Sensitivity analyses after outlier treatment are reported in S3 Table for NavegApp metrics, S4 Table for comparisons involving asymptomatic PSEN1-E280A non-carriers, asymptomatic PSEN1-E280A carriers, and PSEN1-E280A carriers with mild cognitive impairment, and S5 Table for the comparison between healthy older adults and participants with sporadic MCI. Sociodemographics, neuropsychological profile and performance in NavegApp were summarized using descriptive statistics. Socioeconomic status was established and presented in accordance with the National Department of Statistics recommendations for the country [46].

Multiple linear regression models were fitted to explore the effect of carrying the PSEN1-E280A genetic variant on task performance, adjusting for covariates such as education and age. As differential performance in spatial abilities has been described in the literature by sex [47–50], this variable was also considered in the models. When dependent variables described a skewed distribution, a log transformation was used. For those variables with a negatively skewed distribution, the log transformation was applied to the inverse punctuation of the score. Then, Hedges’ g effect size measures and its corresponding confidence interval at 95% were calculated based on the unstandardized regression coefficients by using the *esc* R package [51].

Given the exploratory nature of the study and the number of comparisons conducted (11 outcomes × 4 between-group comparisons, corresponding to 44 effect-size comparisons across Tables 2 and 3), we considered the potential inflation of Type I error. Based on prior theoretical and empirical evidence suggesting that allocentric navigation deficits may emerge early in the Alzheimer’s disease continuum [9,35], Mean Error to Goal from the gHGT was designated as the primary spatial cognition metric for interpretation. The remaining NavegApp metrics were considered exploratory and interpreted with appropriate caution regarding multiple comparisons; readers should consider this when interpreting findings, particularly for effect sizes with confidence intervals near zero. Adjusted confidence intervals were computed across the comparisons in Table 2, and Table 3 as a conservative sensitivity analysis, and are reported in S8 Table.

Multiple linear mixed models with random intercepts for participants were employed to assess the effect of the PSEN1-E280A mutation on allocentric spatial navigation performance. Age, education, and sex were included as covariates in the models. A log transformation was applied to variables with skewed distributions to meet model assumptions, and the exponential function was used to back-transform these values for interpretability, thereby quantifying the mutation’s effect. All the models were computed using the *lme4* R package [52].

The diagnostic accuracy of SC measures was determined by calculating the area under the ROC curve (AUC-ROC), as the response variables were all numerical. An AUC of 0.5 was assumed to indicate a non-informative result, an AUC of 0.5 - 0.7 as low accuracy, an AUC of 0.7-0.9 as moderate accuracy and above 0.9 as high accuracy [53–55].

## Results

Table 1 summarizes the demographic and neuropsychological characteristics of the participants. Across all groups, a higher proportion of females was observed. As anticipated, the mean age varied significantly between groups, with asymptomatic PSEN1-E280A carriers and non-carriers representing the youngest cohorts. In terms of education, most participants completed secondary education, except for the symptomatic PSEN1-E280A carriers group, who reported an average of approximately 9.5 years of schooling. Most participants currently resided in urban areas, reflecting the demographic distribution typical of the region [46]. Regarding the neuropsychological profile, asymptomatic participants—both PSEN1-E280A carriers and non-carriers—performed better across all cognitive assessments. Similarly, older participants without MCI outperformed those with sporadic MCI in cognitive screening tests. Notably, participants with MCI carrying the PSEN1-E280A mutation demonstrated the poorest cognitive performance, with pronounced deficits in memory and visuospatial domains.

**Table 1:** Demographics and neuropsychological profile.

	CU-NC	CU-C	MCI-C	HE-NC	sMCI
	(n = 76)	(n = 99)	(n = 8)	(n = 21)	(n = 22)
**Biological sex assigned to birth**					
Female (%)	45 (59.2%)	68 (68.7%)	6 (75.0%)	13 (61.9%)	12 (54.5%)
Male (%)	31 (40.8%)	31 (31.3%)	2 (25.0%)	8 (38.1%)	10 (45.5%)
**Residence**					
Rural (%)	9 (11.8%)	22 (22.2%)	0 (0%)	4 (19.0%)	2 (9.1%)
Urban (%)	67 (88.2%)	77 (77.8%)	8 (100%)	17 (81.0%)	20 (90.9%)
**Socioeconomic Status**					
High (%)	1 (1.3%)	2 (2.0%)	0 (0%)	4 (19.0%)	8 (36.4%)
Low (%)	72 (94.7%)	96 (97.0%)	8 (100%)	9 (42.9%)	10 (45.5%)
Medium (%)	3 (3.9%)	1 (1.0%)	0 (0%)	8 (38.1%)	4 (18.2%)
**Age**	33.0 [24.8; 40.0]	31.0 [25.0; 39.0]	45.0 [43.3; 47.3]	65.0 [59.0; 68.0]	67.0 [62.5; 71.0]
**Years of education**	12.0 [11.0; 13.0]	11.0 [11.0; 13.0]	9.5 [5.0; 11.0]	13.0 [11.0; 18.0]	15.0 [11.3; 16.0]
**Cognitive Screening**					
MMSE	29.0 [28.0, 30.0]	29.0 [28.0, 30.0]	23.0 [21.8, 23.2]	29.0 [28.0, 29.0]	27.0 [25.0, 28.0]
**Language**					
Semantic Fluency	21.5 [19.0, 26.0]	20.0 [17.0, 23.0]	14.0 [13.2, 17.2]	20.0 [18.0, 21.0]	16.0 [14.2, 17.8]
BNT - 15	13.5 [13.0, 14.0]	13.0 [12.0, 14.0]	12.0 [11.0, 13.0]	14.0 [13.0, 15.0]	14.0 [14.0, 14.0]
**Memory**					
WL Learning	22.0 [20.0, 24.0]	21.0 [18.0, 24.0]	12.0 [10.0, 14.0]	18.0 [16.0, 20.0]	14.0 [12.2, 16.5]
WL Delayed Recall	8.0 [8.0, 9.0]	7.0 [6.0, 9.0]	3.0 [0.8, 3.0]	7.0 [6.0, 8.0]	3.0 [2.2, 5.8]
WL Recognition	10.0 [10.0, 10.0]	10.0 [10.0, 10.0]	7.0 [6.0, 8.0]	10.0 [10.0, 10.0]	8.0 [7.2, 9.0]
RCFT Delayed Recall	25.0 [19.5, 28.0]	19.5 [14.0, 25.0]	5.5 [3.5, 7.4]	17.0 [10.5, 19.5]	7.0 [6.0, 8.4]
**Visuospatial**					
CP Copy	11.0 [10.0, 11.0]	11.0 [10.0, 11.0]	8.0 [7.8, 8.5]	10.0 [10.0, 11.0]	10.0 [10.0, 10.0]
RCFT copy	33.0 [31.9, 35.0]	33.0 [30.0, 34.0]	20.5 [14.2, 23.6]	32.0 [29.0, 34.0]	29.0 [28.2, 32.0]
Raven’s Progressive Matrices	10.0 [9.8, 11.0]	10.0 [9.0, 11.0]	7.0 [5.0, 7.5]	10.0 [8.0, 10.0]	9.0 [8.0, 9.0]
**Processing Speed**					
WAIS-III Digit Symbol	64.0 [52.5, 76.0]	56.0 [46.0, 70.0]	23.0 [20.2, 25.5]	37.5 [30.0, 53.0]	38.0 [32.8, 39.0]
**Executive Functioning**					
Phonemic Fluency (FAS)	35.5 [30.0, 41.0]	32.0 [27.0, 39.0]	28.0 [21.0, 28.0]	36.0 [28.0, 40.0]	39.0 [27.5, 44.0]
WCST - Total Correct	30.0 [24.0, 36.0]	27.5 [21.5, 33.5]	17.0 [14.8, 18.8]	22.0 [13.0, 27.0]	26.0 [18.5, 27.0]
WCST - Total Errors	17.0 [12.0, 22.5]	20.0 [13.5, 26.0]	31.0 [29.2, 33.2]	26.0 [21.0, 35.0]	22.0 [21.0, 28.0]
WCST - Total Categories	4.0 [2.0, 5.0]	3.0 [2.0, 4.5]	1.0 [0.8, 2.0]	3.0 [1.0, 3.0]	2.0 [1.0, 3.0]
WCST - Total Perseverations	10.0 [7.0, 13.2]	13.0 [9.0, 17.0]	21.0 [20.2, 23.5]	18.0 [14.0, 21.0]	15.0 [12.5, 17.5]

*Note. CU-NC = Asymptomatic PSEN1-E280A non-carriers; CU-C = Asymptomatic PSEN1-E280A Carriers; MCI-C = Mild Cognitive Impairment PSEN1-E280A Carriers; sMCI = Sporadic Mild Cognitive Impairment; HE-NC = Cognitively Healthy Elder PSEN1-E280A non-carriers; MMSE = Mini-Mental State Examination; WL = Word List; BNT = Boston Naming Test; RCFT = Rey-Osterrieth Complex Figure; WAIS-III = Wechsler Adult Intelligence Scale, Spanish-III; WCST = Wisconsin Card Sorting Test. Numerical variables are reported as median and interquartile range (IQR)*.

### Spatial cognition performance: PSEN1-E280A participants

Table 2 summarizes participants’ performance on the NavegApp tasks. Additional analyses are provided in the supporting information. Unadjusted bivariate comparisons are reported in S1 Table and estimated marginal means in S2 Table.

**Table 2:** Spatial cognition groups comparison.

	1. CU-NC (n = 76)	*2. CU-C* (n = 99)	*3. MCI-C* (n = 8)	1 vs *2*	1 vs *3*	*2* vs *3*
	Med [IQR]	Med [IQR]	Med [IQR]	Hedges’ g [CI_95%_]	Hedges’ g [CI_95%_]	Hedges’ g [CI_95%_]
**Gamified Hidden Goal Task (gHGT)**						
Mean Path Length	46.60 [37.30, 58.90]	50.60 [39.3, 64.3]	63.00 [54.1, 78.1]	0.21 [-0.08, 0.51]	0.31 [-0.42, 1.04]	-0.16 [-0.88, 0.56]
Mean Path Time	1542.40 [1234.60, 1953.60]	1675.70 [1301.9, 2136.4]	2100.20 [1821.40, 2607.70]	0.21 [-0.09, 0.51]	0.33 [-0.4, 1.06]	-0.14 [-0.86, 0.58]
Mean Error to Goal	15.00 [11.20, 19.10]	17.60 [11.90, 22.70]	36.50 [33.60, 38.70]	0.33 [0.03, 0.63]	1.52 [0.76, 2.28]	1.14 [0.41, 1.88]
**Gamified Mental Rotation Task (gMRT)**						
Total Score	43.00 [33.80, 46.00]	40.00 [30.00, 44.00]	19.50 [16.00, 20.80]	-0.20 [-0.50, 0.10]	-1.04 [-1.79, -0.3]	-1.10 [-1.84, -0.37]
Score 0° Condition	16.00 [16.00, 16.00]	16.00 [15.00, 16.00]	14.50 [13.80, 15.20]	0.25 [-0.05, 0.55]	1.46 [0.71, 2.21]	0.78 [0.05, 1.52]
Score 90° Condition	14.00 [7.80, 16.00]	13.00 [3.50, 15.00]	1.00 [1.00, 1.20]	-0.15 [-0.45, 0.15]	-1.01 [-1.75, -0.26]	-1.05 [-1.79, -0.32]
Score 180° Condition	13.00 [10.00, 14.00]	12.00 [6.00, 14.00]	4.00 [0.80, 4.20]	0.26 [-0.46, 0.98]	0.46 [-0.27, 1.19]	0.59 [-0.13, 1.32]
**Gamified Corsi Task (gCorsi)**						
Span - Forward	5.00 [4.00, 6.00]	5.00 [4.00, 5.00]	0.00 [0.00, 4.00]	-0.20 [-0.50, 0.10]	-0.99 [-1.74, -0.25]	-1.11 [-1.84, -0.37]
Span - Backward	6.00 [4.00, 6.0]	5.00 [3.00, 6.00]	0.00 [0.00, 3.20]	-0.25 [-0.55, 0.05]	-1.08 [-1.83, -0.33]	-1.14 [-1.88, -0.41]
MRT - Forward	3171.20 [2322.9, 4089.5]	3255.90 [2740.2, 3878.7]	3977.40 [3507.90, 4772.2]	0.02 [-0.28, 0.32]	0.55 [-0.18, 1.28]	0.82 [0.09, 1.55]
MRT - Backward	2826.50 [2353.8, 3598.5]	3102.70 [2372.4, 4408.7]	2880.30 [2636.9, 3882.3]	0.15 [-0.15, 0.45]	0.22 [-0.51, 0.95]	-0.25 [-0.97, 0.47]

Note. *CU-NC = Asymptomatic PSEN1-E280A non-carriers; CU-C = Asymptomatic PSEN1-E280A Carriers; MCI-C = Mild Cognitive Impairment PSEN1-E280A Carriers; n = number of participants; Med = Median; IQR = Interquartile Range; CI*_*95%*_ *= Confidence Interval at 95*.

In comparisons between asymptomatic PSEN1-E280A non-carriers and carriers, small differences were observed in both Path length (g = 0.21, 95% CI: [-0.08, 0.51]) and Path time (g = 0.21, 95% CI: [-0.09, 0.51]), with carriers demonstrating longer paths and correspondingly longer completion times. Small differences were detected regarding the mean error to the goal (g = 0.33, 95% CI: [0.03, 0.63]), with PSEN1-E280A carriers performing worse than non-carriers. Additionally, small differences were identified between these groups across all measures of gMRT and gCorsi tasks.

When examining differences between PSEN1-E280A non-carriers and PSEN1-E280A carriers with MCI, small differences were observed in both mean Path Length (g = 0.31, 95% CI: [-0.42, 1.04]) and Path Time (g = 0.33, 95% CI: [-0.40, 1.06]). A large difference was identified in mean error to goal on the gHGT task (g = 1.52, 95% CI: [0.76, 2.28]), with MCI carriers exhibiting higher errors than non-carriers. For the gMRT task, large differences were found across all measures (g = 0.46, 1.49). In the gCorsi task, large differences were observed between groups in span measures and in mean reaction time for the forward condition (g = 0.55, – 1.08), although only a small difference was found in mean reaction time for the backward condition (g = 0.22, 95% CI: [-0.51, 0.95]).

Finally, in comparisons between asymptomatic PSEN1-E280A carriers and MCI PSEN1-E280A carriers, small differences were observed in mean Path Length (g = -0.16, 95% CI: [-0.88, 0.56]) and Path Time (g = -0.14, 95% CI: [-0.86, 0.58]), while large differences were found in mean error to goal on gHGT task (g = 1.14, 95% CI: [0.41, 1.88]). Large differences were found in the scores of the 0° condition (g = 1.14, 95% CI: [0.41, 1.88]) and 90° condition (g = 1.14, 95% CI: [0.41, 1.88]) of gMRT. Similarly, large differences were observed in gCorsi metrics (g = 0.82, -1.14), except for mean reaction time in the backward condition (g = 0.25, 95% CI: [-0.97, 0.47]).

Table 3 summarizes the performance and comparison of healthy older participants and those with sporadic MCI across the SC measures derived from NavegApp. As in previous analyses, Hedges’ g values indicate the standardized differences between groups, adjusted for covariates such as sex, education, and age.

**Table 3:** Spatial cognition performance.

	1. HE-NC	2. sMCI	1 vs 2
	(n = 21) Med [IQR]	(n = 22) Med [IQR]	Hedges’ g [CI_95%_]
**Gamified Hidden Goal Task (gHGT)**			
Mean Path Distance	52.1 [45.0, 65.8]	75.8 [58.4, 84.1]	0.95 [0.32, 1.58]
Mean Path Time	1725.3 [1508.4, 2179.9]	2523.9 [1943.0, 2821.4]	0.97 [0.34, 1.61]
Mean Error to Goal	17.9 [11.1, 27.9]	23.6 [18.3, 27.4]	0.53 [-0.08, 1.14]
**Gamified Mental Rotation Task (gMRT)**			
Total Score	42.0 [27.0, 45.0]	33.5 [29.0, 42.8]	-0.13 [-0.73, 0.47]
Score 0° Condition	16.0 [15.0, 16.0]	16.0 [15.0, 16.0]	0.49 [-0.12, 1.09]
Score 90° Condition	14.0 [3.0, 15.0]	7.50 [1.2, 13.5]	-0.36 [-0.96, 0.24]
Score 180° Condition	11.0 [5.0, 15.0]	12.0 [10.2, 14.0]	-0.20 [-0.80, 0.40]
**Gamified Corsi Task (gCorsi)**			
Span – Forward	5.0 [4.0, 6.0]	4.0 [4.0, 5.0]	-0.32 [-0.92, 0.28]
Span – Backward	5.0 [4.0, 5.0]	4.0 [3.0, 5.0]	-0.31 [-0.91, 0.29]
MRT – Forward	3257.7 [2834.8, 4581.5]	4539.0 [3578.7, 4863.0]	0.43 [-0.18, 1.03]
MRT – Backward	3545.6 [2553.8, 4378.7]	3829.0 [2631.6, 5054.5]	0.19 [-0.41, 0.79]

*Note. sMCI = Sporadic Mild Cognitive Impairment; Med = Median; IQR = Interquartile range; CI*_*95%*_ *= Confidence interval at 95%*.

When comparing healthy older participants to those with sporadic MCI, differences were observed in the gHGT metrics. Sporadic MCI participants exhibited longer path distances (g = 0.95, 95% CI [0.32, 1.58]) and completion times (g = 0.97, 95% CI [0.34, 1.61]) compared to healthy elders. In the gMRT, no substantial differences were found in the total score (g = -0.13, 95% CI [-0.73, 0.47]), and only small differences were detected across all conditions. For the gCorsi task, small differences were noted in span measures for both forward (g = -0.32, 95% CI [-0.92, 0.28]) and backward (g = -0.31, 95% CI [-0.91, 0.29]) conditions. Similarly, small differences in mean reaction time were observed in the forward condition (g = 0.43, 95% CI [-0.18, 1.03]). The results for the gMRT and gCorsi tasks indicate the observed effect is uncertain, as the confidence interval includes zero.

### Linear mixed models

Linear mixed-effects models with subject-level random effects were adjusted to compare groups performance on the gHGT. Each model included genetic status, age, sex, education, and trial number as covariates. For analysis involving asymptomatic participants and PSEN1-E280A carriers with MCI, PSEN1-E280A non-carriers were used as the reference group. A log transformation was applied to address the positively skewed distribution of the variables. Models presented in Table 4 were selected based on Akaike Information Criterion (AIC), theoretical plausibility, and parsimony. Residual diagnostic plots for the mixed-effects models are shown in S2–S7 Figs. For models comparing asymptomatic participants and PSEN1-E280A carriers, plots are provided for path length in S2 Fig, path time in S3 Fig, and mean error to goal in S4 Fig. For models comparing healthy older adults and participants with sporadic MCI, plots are provided for path length in S5 Fig, path time in S6 Fig, and mean error to goal in S7 Fig.

**Table 4:** Linear mixed effect models for the gHGT.

	Path Length		Path Time		Error to Goal	
*Predictors*	*Estimates*	*CI*	*Estimates*	*CI*	*Estimates*	*CI*
**PSEN1-E280A groups**						
(Intercept)	3.69	3.39 – 3.99	7.19	6.89 – 7.49	2.64	2.26 – 3.02
Group: E280A carrier	0.09	-0.03 – 0.21	0.09	-0.03 – 0.21	0.13	0.01 – 0.26
Group: MCI E280A carrier	0.24	-0.05 – 0.54	0.26	-0.04 – 0.56	0.70	0.39 – 1.01
Education	-0.02	-0.03 – -0.00	-0.02	-0.03 – -0.00	-0.02	-0.04 – 0.00
Age	0.01	0.00 – 0.01	0.01	0.00 – 0.01	0.01	0.00 – 0.02
Sex: Female	0.01	-0.09 – 0.11	0.01	-0.09 – 0.11	0.22	0.10 – 0.35
Trial	0.02	0.01 – 0.03	0.02	0.01 – 0.03	-0.09	-0.10 – -0.07
E280A carrier: Trial	-0.01	-0.02 – 0.01	-0.01	-0.02 – 0.01	–	–
MCI E280A carrier: Trial	-0.08	-0.11 – -0.04	-0.08	-0.12 – -0.04	–	–
**Random Effects**						
σ^2^	0.10		0.11		0.42	
ICC	0.47		0.46		0.21	
Marginal R^2^ / Conditional R^2^	0.059/ 0.498		0.058/ 0.494		0.150/ 0.328	
**Healthy Elder vs Sporadic MCI**						
(Intercept)	4.44	3.60 – 5.27	7.93	7.08 – 8.78	1.79	0.69 – 2.89
Group: Sporadic MCI	0.3	0.11 – 0.48	0.31	0.12 – 0.49	0.29	0.05 – 0.53
Education	-0.01	-0.03 – 0.01	-0.01	-0.03 – 0.01	-0.02	-0.05 – 0.01
Age	-0.01	-0.02 – 0.00	-0.01	-0.02 – 0.00	0.02	0.01 – 0.04
Sex: Female	0.02	-0.17 – 0.21	0.03	-0.17 – 0.22	0.24	-0.02 – 0.49
Trial	0.02	0.01 – 0.03	0.02	0.00 – 0.03	-0.06	-0.09 – -0.03
**Random Effects**						
σ^2^	0.10		0.10		0.36	
ICC	0.44		0.44		0.23	
Marginal R^2^ / Conditional R^2^	0.132/ 0.512		0.134/ 0.511		0.192/ 0.381	

*Note: CI = 95% confidence interval; PSEN1-E280A = Presenilin-1 E280A mutation; MCI = Mild Cognitive Impairment; ICC = intraclass correlation coefficient; σ² = residual variance; R² = coefficient of determination*.

Compared to asymptomatic non-carriers, asymptomatic PSEN1-E280A carriers demonstrated poorer performance across all task metrics. Specifically, carriers exhibited path lengths 9% longer (β = 0.09, 95% CI [-0.03, 0.21]), completion times 9% higher (β = 0.09, 95% CI [-0.03, 0.21]), and errors to the goal 14% higher (β = 0.13, 95% CI [0.01, 0.26]). These impairments were more pronounced in MCI PSEN1-E280A carriers, who showed path lengths 27% longer (β = 0.24, 95% CI [-0.05, -0.54]), completion times 26% higher (β = 0.24, 95% CI [-0.04, -0.56]), and errors to the goal 101% higher (β = 0.70, 95% CI [0.39, 1.01]). Similarly, participants with sporadic MCI exhibited significantly impaired performance compared to healthy elderly controls, with path lengths 35% longer (β = 0.30, 95% CI [0.11, 0.48]), completion times 36% higher (β = 0.31, 95% CI [0.12, 0.49]), and errors to the goal 34% higher (β = 0.29, 95% CI [0.05, 0.53]).

### Diagnostic accuracy of spatial cognition measures

Table 5 summarizes the results of the diagnostic accuracy analyses for different groups of participants. Given the similar performance observed between asymptomatic PSEN1-E280A carriers and non-carriers, the diagnostic accuracy analyses were conducted by combining these individuals into a single asymptomatic group. Results stratified by genetic status are presented in S6 Table. Complementary diagnostic accuracy metrics such as sensitivity, specificity, and predictive values, are provided in S7 Table.

**Table 5:** Diagnostic accuracy of NavegApp.

	CU-C Vs. CU-NC AUC [CI_95%_]	Asymptomatic Vs MCI-C AUC [CI_95%_]	HE-NC Vs. sMCI AUC [CI_95%_]
**Gamified Hidden Goal Task (gHGT)**			
Mean Path Distance	0.57 [0.48, 0.65]	0.63 [0.36, 0.91]	0.77 [0.62, 0.91]
Mean Path Time	0.57 [0.48, 0.65]	0.64 [0.37, 0.91]	0.77 [0.62, 0.92]
Mean Error to Goal	0.60 [0.52, 0.69]	0.94 [0.85, 1.00]	0.65 [0.47, 0.85]
**Gamified Mental Rotation Task (gMRT)**			
Total Score	0.59 [0.51, 0.68]	0.91 [0.84, 0.98]	0.56 [0.38, 0.75]
Score 0° Condition	0.56 [0.50, 0.62]	0.78 [0.60, 0.97]	0.59 [0.44, 0.73]
Score 90° Condition	0.56 [0.48, 0.65]	0.86 [0.79, 0.92]	0.64 [0.46, 0.81]
Score 180° Condition	0.59 [0.51, 0.68]	0.84 [0.69, 0.98]	0.45 [0.27, 0.63]
**Gamified Corsi Task (gCorsi)**			
Span - Forward	0.57 [0.49, 0.65]	0.80 [0.68, 0.93]	0.63 [0.47, 0.8]
Span - Backward	0.59 [0.50, 0.67]	0.83 [0.73, 0.92]	0.62 [0.45, 0.79]
RT - Forward	0.52 [0.43, 0.61]	0.71 [0.56, 0.87]	0.67 [0.50, 0.84]
RT - Backward	0.56 [0.47, 0.64]	0.51 [0.28, 0.73]	0.56 [0.39, 0.74]

*Note. CU-C = Asymptomatic PSEN1-E280A Carriers; CU-NC = Asymptomatic PSEN1-E280A non-carriers; MCI-C = Mild Cognitive Impairment PSEN1-E280A Carriers; sMCI = Sporadic Mild Cognitive Impairment; HE-NC = Cognitively Healthy Elder PSEN1-E280A non-carriers; RT = Reaction Time*.

For gHGT-related metrics, the mean path length and path time showed limited diagnostic value for differentiating asymptomatic PSEN1-E280A carriers from non-carriers, as neither metric demonstrated informative discriminatory capacity (AUC-ROC = 0.57). In contrast, the mean error to goal exhibited a low diagnostic accuracy, with an AUC-ROC of 0.60 (CI 95%: 0.52–0.69). When comparing asymptomatic participants with MCI PSEN1-E280A carriers, mean error to goal demonstrated excellent diagnostic accuracy, achieving an AUC-ROC range of 0.94 to 0.97. In contrast, path length and path time showed a low diagnostic accuracy for distinguishing between these groups, with AUC-ROC values ranging from 0.63 to 0.69. For the comparison between participants with sporadic MCI and healthy controls, mean error to goal exhibited low diagnostic accuracy (AUC-ROC = 0.65, 95%CI [0.47, 0.85]). In contrast, path length and path completion time showed acceptable diagnostic accuracy, with an AUC-ROC of 0.77.

For the gMRT metrics, diagnostic accuracy was low for differentiating asymptomatic individuals, with AUC-ROC values ranging from 0.56 to 0.59, indicating low discrimination. However, in comparisons between asymptomatic individuals and PSEN1-E280A carriers with MCI, all metrics showed moderate to high diagnostic accuracy, with AUC-ROC values from 0.78 to 0.91. For the comparison between sporadic MCI participants and age-matched controls, non-informative diagnostic accuracy was observed for all the task metrics (AUC-ROC = 0.54 - 0.59), and only the Total Score for the 90° condition demonstrated low diagnostic accuracy, with an AUC-ROC of 0.64 (95% IC: [0.46, 0.81]).

For the gCorsi metrics, diagnostic accuracy was low for asymptomatic participants, with AUC-ROC values between 0.52 and 0.59, indicating non-informative discrimination. In comparing asymptomatic individuals to MCI PSEN1-E280A carriers, all metrics except the mean reaction time for the backward condition showed moderate diagnostic accuracy, with AUC-ROC values ranging from 0.70 to 0.83. Conversely, in comparisons between sporadic MCI participants and age-matched healthy controls, all metrics showed low diagnostic accuracy, with AUC-ROC values between 0.56 and 0.67.

## Discussion

With the global population aging and limited access to effective pharmacological treatments, there is an urgent need for accessible methods to detect AD in its earliest stages, enabling preventive interventions that may delay symptom onset [56]. In this study, we evaluated the diagnostic accuracy of NavegApp, a novel, serious game-based platform, for distinguishing individuals at different stages of AD. NavegApp includes three gamified tasks selected based on theoretical and empirical evidence to represent core components of SC potentially sensitive to cognitive changes during the preclinical and prodromal stages of AD [13,17,57,58]. Our findings reveal that SC metrics demonstrate variable diagnostic accuracy depending on the comparison groups, reflecting the progressive impact of AD on brain regions involved in SC. The implications of these findings for early AD detection and future research are discussed in detail below.

Over the last decade, numerous studies have highlighted the potential of SC as a source of cognitive markers for the early detection of AD [18]. Most research has focused on comparing spatial navigation performance, assessed through VR and digital interfaces, between symptomatic individuals (e.g., those with MCI or early dementia) and cognitively healthy controls [18]. In these contexts, VR-based navigation tasks have proven accurate in distinguishing between groups, with cognitively impaired individuals consistently showing reduced performance across various spatial tasks and paradigms [18]. However, fewer studies have investigated the diagnostic accuracy of SC tasks for identifying cognitively healthy individuals based on biomarkers, revealing a limited capacity of these tasks to differentiate between such groups [18].

In this study, we evaluated the capacity of NavegApp to distinguish individuals at different stages of AD. To our knowledge, this is the first study to assess the diagnostic accuracy of digital, gamified SC tasks in a sample that includes individuals carrying a causative mutation for early-onset AD. This provides valuable evidence of the utility of SC tasks for distinguishing individuals at the asymptomatic stage of the disease [18,57,59].

Small differences were observed across all SC metrics when comparing asymptomatic participants. However, PSEN1-E280A carriers demonstrated larger impairments in the gHGT, characterized by higher errors to goal, longer path lengths, and increased completion times. These findings align with previous studies reporting alterations in spatial memory [60] and cognitive mapping tasks [61] in the preclinical AD stage and support the notion that individuals at risk of developing AD exhibit diminished allocentric spatial navigation performance, aligning with the expected pattern of disease progression [18,22,61]. Nevertheless, the small magnitude and high uncertainty of these differences limit the diagnostic accuracy of SC tasks included in NavegApp, similar to prior studies comparing individuals based on biomarker status [61]. Because the AUC-ROC reflects the ability to distinguish between groups [62], small performance differences yield non-informative or low diagnostic accuracy in asymptomatic individuals.

When comparing asymptomatic participants with MCI PSEN1-E280A carriers, moderate to large differences were observed in SC metrics assessed by NavegApp. Notably, performance on the gHGT was highly impaired (g = 1.14 – 1.52), indicating disruptions in spatial processing using external landmarks. This finding aligns with the expected pattern of cognitive impairment progression in AD [9]. Consistent with this, previous studies have reported severe impairments in allocentric spatial navigation during the prodromal stage of AD [63–66]. While these studies demonstrate adequate discriminative capacity for distinguishing symptomatic individuals from cognitively healthy controls [18], they often rely on VR-based cognitive assessments, which require substantial physical space and specialized equipment. By contrast, NavegApp provides a gamified interface with automated task execution, streamlining the assessment process [23].

As hypothesized, symptomatic individuals exhibited significant impairments in other SC processes. When comparing asymptomatic participants to MCI PSEN1-E280A carriers, large differences were observed across all gMRT metrics (g = 0.85 – 1.17), indicating substantial deficits in mental rotation abilities. These findings, combined with the cognitive profiles of the participants, suggest more pronounced cognitive impairments in individuals with early-onset AD. Our results differ from previous studies, which reported differences in the 90° and 180° conditions when comparing individuals with early dementia to healthy controls [12]. Discrepancies in demographic variables, such as education or access to technology, may account for these differences. Further replication studies involving diverse populations are needed to validate these findings.

Regarding spatial memory assessed by NavegApp, our findings indicate that the gCorsi task demonstrates adequate discriminative capacity for distinguishing individuals at the prodromal stage of early-onset AD from cognitively healthy individuals (AUC-ROC = 0.70–0.85). However, its ability to differentiate between healthy elderly participants and those with sporadic MCI is limited (AUC-ROC = 0.56 – 0.67). Previous studies have consistently reported impairments in visuospatial memory among individuals with MCI and mild dementia, showing differences compared to healthy controls [36,67]. In this context, our results align with the existing literature and support the notion that impairments in visuospatial working memory tend to manifest later in the AD continuum [36].

The findings of this study underscore the potential of SC as a valuable source of cognitive markers, not only for early identification but also for tracking disease progression. Including individuals at risk for early-onset AD, such as PSEN1-E280A carriers, provided unique insights into the earliest cognitive changes in individuals destined to develop AD [59]. The observed differences among asymptomatic participants suggest that impairments in navigation abilities may emerge in the early stages of the disease [60,68] when traditional paper-and-pencil neuropsychological tests are insufficient to detect objective cognitive impairments [68]. Conversely, the absence of substantial differences in gMRT and gCorsi tasks when comparing asymptomatic participants suggests that hippocampal-dependent processes, as measured by tasks like gHGT, hold greater promise as early cognitive markers for identifying individuals at risk for AD.

### Implications for research

As the demand for accurate cognitive markers to detect the earliest changes along the AD continuum rises, evaluating the diagnostic accuracy of emerging digital tools across different disease stages becomes increasingly critical. Preliminary findings suggest NavegApp may distinguish individuals in prodromal stages of AD from asymptomatic individuals. However, this evidence should be interpreted cautiously, as the small sample size of the PSEN1-E280A MCI group (n = 8) precludes definitive conclusions and underscores the need for substantially larger validation samples. Taken together, these preliminary results indicate that NavegApp represents a promising, yet still exploratory, alternative to existing assessment approaches, with the added benefit of a gamified digital format.

Over the past decade, digital technologies have demonstrated considerable advantages in automating cognitive assessments, enabling the exploration of disease progression through innovative approaches [18]. SC-related processes, guided by models of AD progression, have emerged as promising targets for developing cognitive markers. Our findings support this perspective, showing that allocentric spatial navigation is affected during the preclinical stage of AD in otherwise cognitively healthy individuals [60,61,69]. Using a gamified allocentric navigation task, we observed small but measurable differences between PSEN1-E280A carriers and non-carriers. Notably, the mean age of our participants situates them approximately ten years before the expected symptom onset, challenging the traditional view of the preclinical stage as devoid of cognitive changes [6].

The use of digital tools in video game formats has been proposed as a feasible approach for cognitive assessment, particularly for asymptomatic or early symptomatic stages of neurodegenerative diseases like AD [70]. Despite their advantages, serious games and gamified tasks remain underutilized in cognitive assessment [18]. Our findings underscore the potential of SG-based tools to effectively capture relevant SC data, offering unique insights into the cognitive performance of at-risk populations [69,71].

These results highlight the potential of gamified digital platforms for advancing cognitive assessment, particularly for identifying and monitoring at-risk individuals in the preclinical and prodromal stages of AD [24,70,72]. Future research should focus on expanding the application of serious games across diverse populations and integrating these tools into broader frameworks for early detection and intervention, ultimately contributing to the global effort to combat AD.

### Considerations for the cognitive assessment of the PSEN1-E280A cohort

The near-certain progression to AD in PSEN1-E280A carriers offers a unique and valuable model to study cognitive changes early in the disease process, particularly those linked to its natural progression [26]. Research indicates that Tau protein elevations in cerebrospinal fluid appear around age 24, and amyloid deposits become detectable via PET scans by age 28 in this cohort [73]. Periodic cognitive assessments have identified alterations in memory and other cognitive domains during both asymptomatic and prodromal stages. Differences in verbal memory have been observed up to 14 years before symptom onset, including impairments in word-list learning tasks from the CERAD-Col battery [32]. While deficits in language, executive function, and attention primarily appear in symptomatic stages [59,73], early changes in visuospatial abilities have been reported in associative memory-binding tasks [28,74], even when other neuropsychological tests, such as the RCFT or Raven’s progressive matrices, fail to show differences [59]. These findings highlight the importance of targeted cognitive markers for identifying subtle changes during the preclinical stage of AD in cohorts with genetic risk for early-onset AD.

In this context, NavegApp provides a novel approach to cognitive assessment in the PSEN1-E280A cohort. While previous research indicates limited impairments in the visuospatial domain across the AD continuum [59], our findings reveal that even asymptomatic individuals show diminished performance in SC-based tasks using a digital, gamified tool. These results highlight the potential of NavegApp as a valuable instrument for monitoring clinical progression in cohorts at risk for AD and warrant further research to demonstrate its feasibility in clinical and populational settings.

### Limitations

While the findings of this study are promising, several limitations must be acknowledged to ensure a balanced interpretation of the results. First, group size disparities emerged due to the rapid progression of PSEN1-E280A carriers from the prodromal to early dementia stages, limiting the availability of participants in the prodromal phase [27]. The small sample size of the PSEN1-E280A MCI group reflects the limited availability of individuals at this stage and the rapid progression characteristic of this mutation, which substantially narrows the window for recruitment during mild cognitive impairment. This uneven distribution may have influenced the robustness of our conclusions for this subgroup. Additionally, although participant classification relied on rigorous clinical evaluations conducted by trained personnel, the pronounced cognitive decline in PSEN1-E280A carriers could have introduced bias, potentially overestimating the diagnostic accuracy of NavegApp.

In addition, the study did not pre-register a primary outcome, and family-wise error rate corrections were not applied to the main analyses. Therefore, the confidence intervals reported in Tables 2 and 3 are unadjusted for the comparisons conducted, which may increase the probability that some reported effects, particularly those with small magnitudes in asymptomatic comparisons, represent false-positive findings. These results should therefore be interpreted as exploratory and hypothesis-generating rather than confirmatory evidence.

The generalizability of these findings is limited by the genetic, sociocultural, geographic, and socioeconomic characteristics of the study population, as well as by contextual factors related to technology use and digital literacy. Accordingly, the results should be interpreted as context-specific evidence and should not be assumed to generalize to other populations, including other Spanish-speaking groups or individuals with different genetic risk profiles. Further studies should focus on evaluating the tool’s performance in more diverse populations and settings to enhance its generalizability. Longitudinal studies tracking individuals from the preclinical stage of AD could provide critical insights into the impact of age on diagnostic accuracy and the progression of NavegApp metrics across the AD continuum.

Although NavegApp holds promise as a cognitive assessment digital tool, it remains in the early stages of development. Further research is essential to establish its diagnostic accuracy across the full spectrum of AD and ensure its reliability as a tool for early detection and disease monitoring. Therefore, the construct validity of NavegApp’s measures requires further investigation to validate the underlying cognitive constructs they assess and to address uncertainties about the validity of SC metrics. Establishing these connections would strengthen confidence in the tool and facilitate correlations between NavegApp outcomes, biomarkers, and other validated SC assessments. Such evidence could form the foundation for developing more sophisticated measures of SC in the context of AD.

Lastly, our analytical approach accounted for covariates and confounders under assumptions of normality and other conditions, which, while supported by the literature, may only sometimes be held in practice. This highlights the need for further studies to assess the robustness of our findings under alternative methodological frameworks.

## Conclusion

In conclusion, SG-based cognitive assessment tools represent a promising and scalable approach for addressing current challenges in AD detection at both individual and community levels [21,70,75]. Our findings demonstrate the feasibility and potential of NavegApp as a valuable tool for assessing cognitive function across diverse populations, particularly for identifying subtle changes associated with the early stages of AD. By leveraging technological innovation, this research contributes to the growing evidence supporting digital solutions in neuropsychological assessment. We aim to inspire further exploration of gamified approaches that can enhance early detection, enable personalized interventions, and mitigate the devastating impacts of AD.

## Supporting information

S1 Table Bivariate comparisons, including statistical significance and effect size metrics. (DOCX)

S2 Table Marginal means derived from linear mixed effects models. (DOCX)

S3 Table Sensitivity analyses for NavegApp metrics after outlier correction. (DOCX)

S4 Table Groups comparison involving asymptomatic young individuals and PSEN1-E280A carriers after outlier correction. (DOCX)

S5 Table Groups comparison involving healthy elders and sporadic MCI participants after outlier correction. (DOCX)

S6 Table Diagnostic accuracy analyses by genetic status. (DOCX)

S7 Table Complementary diagnostic accuracy metrics. (DOCX)

S8 Table Adjusted confidence intervals for groups comparisons. (DOCX)

S1 Fig Distribution of NavegApp variables before and after missing-data imputation. (DOCX)

S2 Fig Residual diagnostics for the path length mixed-effects model in PSEN1-E280A group comparisons. (DOCX)

S3 Fig Residual diagnostics for the path length mixed-effects model comparing healthy older adults and sporadic MCI participants. (DOCX)

S4 Fig Residual diagnostics for the path time mixed-effects model in PSEN1-E280A group comparisons. (DOCX)

S5 Fig Residual diagnostics for the path time mixed-effects model comparing healthy older adults and sporadic MCI participants. (DOCX)

S6 Fig Residual diagnostics for the mean error to goal mixed-effects model in PSEN1-E280A group comparisons. (DOCX)

S7 Fig Residual diagnostics for the mean error to goal mixed-effects model comparing healthy older adults and sporadic MCI participants. (DOCX)
