# Supplementary material for: NavegApp, a serious game for assessing spatial cognition: Diagnostic accuracy in preclinical and prodromal Alzheimer’s disease
Source: PLOS Digit Health. 2026 Jul 10;5(7):e0001521. doi: 10.1371/journal.pdig.0001521 (PMC13354000; doi:10.1371/journal.pdig.0001521)
Supplement: S1 Table — (DOCX) [file pdig.0001521.s001.docx]

## S1 Table. Bivariate comparisons.

The following section presents the bivariate comparisons between groups. The rank-biserial correlation was used to measure the effect size, as this method is well-suited for variables with skewed distributions or the presence of outliers.

|  | **Task** | **Group 1** | **Group 2** | **Mann-Whitney U test** | | **Rank biserial correlation** | |
| --- | --- | --- | --- | --- | --- | --- | --- |
|  |  |  |  | **Statistic** | **p-value** | **rrb** | **CI_95%_** |
| **Gamified Hidden Goal Task** | | |  |  |  |  |  |
|  | Mean Path Length | PSEN1-E280A Carrier | PSEN1-E280A Non-carrier | 4278 | 0.209 | 0.12 | [0.01, 0.28] |
|  |  | PSEN1-E280A Carrier | MCI PSEN1-E280A Carrier | 289.5 | 0.209 | 0.12 | [0.01, 0.35] |
|  |  | PSEN1-E280A Non-carrier | MCI PSEN1-E280A Carrier | 194 | 0.209 | 0.18 | [0.01, 0.42] |
|  |  | Healthy Elder | Sporadic MCI | 108.5 | 0.012 | 0.45 | [0.19, 0.68] |
|  | Mean Path Time | PSEN1-E280A Carrier | PSEN1-E280A Non-carrier | 4278 | 0.251 | 0.12 | [0.01, 0.26] |
|  |  | PSEN1-E280A Carrier | MCI PSEN1-E280A Carrier | 284 | 0.251 | 0.13 | [0.01, 0.35] |
|  |  | PSEN1-E280A Non-carrier | MCI PSEN1-E280A Carrier | 190 | 0.251 | 0.19 | [0.01, 0.43] |
|  |  | Healthy Elder | Sporadic MCI | 106 | 0.008 | 0.46 | [0.19, 0.69] |
|  | Mean Error to Goal | PSEN1-E280A Carrier | PSEN1-E280A Non-carrier | 4533 | 0.041 | 0.18 | [0.04, 0.31] |
|  |  | PSEN1-E280A Carrier | MCI PSEN1-E280A Carrier | 51 | 0.000135 | 0.40 | [0.23, 0.52] |
|  |  | PSEN1-E280A Non-carrier | MCI PSEN1-E280A Carrier | 20 | 0.0000624 | 0.47 | [0.30, 0.60] |
|  |  | Healthy Elder | Sporadic MCI | 163 | 0.101 | 0.25 | [0.02, 0.54] |
| **Gamified Mental Rotation Task** | | |  |  |  |  |  |
|  | Total Score | PSEN1-E280A Carrier | PSEN1-E280A Non-carrier | 3070.5 | 0.074 | 0.13 | [0.02, 0.30] |
|  |  | PSEN1-E280A Carrier | MCI PSEN1-E280A Carrier | 719 | 0.000393 | 0.37 | [0.23, 0.50] |
|  |  | PSEN1-E280A Non-carrier | MCI PSEN1-E280A Carrier | 566 | 0.00026 | 0.44 | [0.27, 0.56] |
|  |  | Healthy Elder | Sporadic MCI | 260.5 | 0.48 | 0.11 | [0.01, 0.42] |
|  | Score 0° Condition | PSEN1-E280A Carrier | PSEN1-E280A Non-carrier | 3315.5 | 0.1180000 | 0.14 | [0.01, 0.28] |
|  |  | PSEN1-E280A Carrier | MCI PSEN1-E280A Carrier | 619.0 | 0.0030000 | 0.32 | [0.09, 0.51] |
|  |  | PSEN1-E280A Non-carrier | MCI PSEN1-E280A Carrier | 502.0 | 0.0000696 | 0.47 | [0.17, 0.70] |
|  |  | Healthy Elder | Sporadic MCI | 271.5 | 0.2480000 | 0.18 | [0.01, 0.48] |
|  | Score 90° Condition | PSEN1-E280A Carrier | PSEN1-E280A Non-carrier | 3291.5 | 0.15300 | 0.11 | [0.01, 0.25] |
|  |  | PSEN1-E280A Carrier | MCI PSEN1-E280A Carrier | 678.5 | 0.00200 | 0.33 | [0.20, 0.44] |
|  |  | PSEN1-E280A Non-carrier | MCI PSEN1-E280A Carrier | 556.0 | 0.00042 | 0.42 | [0.27, 0.56] |
|  |  | Healthy Elder | Sporadic MCI | 295.0 | 0.15300 | 0.24 | [0.01, 0.54] |
|  | Score 180° Condition | PSEN1-E280A Carrier | PSEN1-E280A Non-carrier | 3072.5 | 0.073 | 0.16 | [0.02, 0.30] |
|  |  | PSEN1-E280A Carrier | MCI PSEN1-E280A Carrier | 662.5 | 0.005 | 0.31 | [0.13, 0.44] |
|  |  | PSEN1-E280A Non-carrier | MCI PSEN1-E280A Carrier | 524.0 | 0.003 | 0.37 | [0.19, 0.52] |
|  |  | Healthy Elder | Sporadic MCI | 206.5 | 0.557 | 0.09 | [0.00, 0.38] |
| **Gamified Corsi Task** | | |  |  |  |  |  |
|  | Span - Forward | PSEN1-E280A Carrier | PSEN1-E280A Non-carrier | 3236.0 | 0.121 | 0.12 | [0.01, 0.27] |
|  |  | PSEN1-E280A Carrier | MCI PSEN1-E280A Carrier | 637.5 | 0.009 | 0.29 | [0.12, 0.42] |
|  |  | PSEN1-E280A Non-carrier | MCI PSEN1-E280A Carrier | 516.0 | 0.004 | 0.36 | [0.20, 0.50] |
|  |  | Healthy Elder | Sporadic MCI | 293.0 | 0.121 | 0.24 | [0.01, 0.52] |
|  | Span - Backward | PSEN1-E280A Carrier | PSEN1-E280A Non-carrier | 3114.5 | 0.090 | 0.15 | [0.01, 0.30] |
|  |  | PSEN1-E280A Carrier | MCI PSEN1-E280A Carrier | 653.5 | 0.005 | 0.30 | [0.16, 0.42] |
|  |  | PSEN1-E280A Non-carrier | MCI PSEN1-E280A Carrier | 538.5 | 0.001 | 0.40 | [0.25, 0.53] |
|  |  | Healthy Elder | Sporadic MCI | 285.0 | 0.184 | 0.20 | [0.01, 0.50] |
|  | MRT -Forward | PSEN1-E280A Carrier | PSEN1-E280A Non-carrier | 3895.0 | 0.605 | 0.04 | [0.00, 0.19] |
|  |  | PSEN1-E280A Carrier | MCI PSEN1-E280A Carrier | 196.0 | 0.174 | 0.18 | [0.04, 0.31] |
|  |  | PSEN1-E280A Non-carrier | MCI PSEN1-E280A Carrier | 161.0 | 0.174 | 0.19 | [0.04, 0.32] |
|  |  | Healthy Elder | Sporadic MCI | 146.0 | 0.174 | 0.29 | [0.03, 0.56] |
|  | MRT - Backward | PSEN1-E280A Carrier | PSEN1-E280A Non-carrier | 3953.0 | 0.832 | 0.10 | [0.00, 0.25] |
|  |  | PSEN1-E280A Carrier | MCI PSEN1-E280A Carrier | 330.0 | 0.943 | 0.01 | [0.00, 0.20] |
|  |  | PSEN1-E280A Non-carrier | MCI PSEN1-E280A Carrier | 229.0 | 0.943 | 0.06 | [0.00, 0.27] |
|  |  | Healthy Elder | Sporadic MCI | 201.0 | 0.943 | 0.11 | [0.01, 0.41] |

*Note. rrb = Rank Biserial Correlation, CI_95%_ = Confidence Interval at 95%. All p-values reported have been adjusted by using the Benjamini-Hochberg method.*
