# Supplementary material for: NavegApp, a serious game for assessing spatial cognition: Diagnostic accuracy in preclinical and prodromal Alzheimer’s disease
Source: PLOS Digit Health. 2026 Jul 10;5(7):e0001521. doi: 10.1371/journal.pdig.0001521 (PMC13354000; doi:10.1371/journal.pdig.0001521)
Supplement: S2 Table — (DOCX) [file pdig.0001521.s002.docx]

## S2 Table. Marginal Means.

Marginal means were derived from the multiple linear regression models and represent the adjusted group differences in the outcome variable, accounting for the effects of covariates such as sex, age, and education.

| **Variable** | **Groups** | **Mean** | **SE** | **95% CI** |
| --- | --- | --- | --- | --- |
| Mean Path Length | PSEN1-E280A Non-carrier | 49.27 | 1.65 | [46.01, 52.53] |
|  | PSEN1-E280A Carrier | 52.60 | 1.50 | [49.63, 55.57] |
|  | PSEN1-E280A Carrier | 53.34 | 1.75 | [49.88, 56.80] |
|  | PSEN1-E280A Carrier MCI | 50.56 | 6.36 | [37.94, 63.17] |
|  | PSEN1-E280A Non-carrier | 49.61 | 1.92 | [45.79, 53.43] |
|  | PSEN1-E280A Carrier MCI | 54.91 | 6.26 | [42.46, 67.37] |
|  | Healthy Elder | 55.22 | 4.25 | [46.61, 63.82] |
|  | Sporadic MCI | 73.14 | 4.09 | [64.86, 81.41] |
| Mean Path Time | PSEN1-E280A Non-carrier | 1632.96 | 55.36 | [1523.68, 1742.23] |
|  | PSEN1-E280A Carrier | 1745.14 | 50.35 | [1645.75, 1844.53] |
|  | PSEN1-E280A Carrier | 1770.00 | 58.52 | [1653.93, 1886.06] |
|  | PSEN1-E280A Carrier MCI | 1687.50 | 213.21 | [1264.60, 2110.40] |
|  | PSEN1-E280A Non-carrier | 1644.35 | 64.15 | [1516.66, 1772.05] |
|  | PSEN1-E280A Carrier MCI | 1834.72 | 209.32 | [1418.09, 2251.36] |
|  | Healthy Elder | 1831.10 | 142.55 | [1542.52, 2119.69] |
|  | Sporadic MCI | 2444.22 | 137.14 | [2166.60, 2721.84] |
| Mean Error to Goal | PSEN1-E280A Non-carrier | 2.66 | 0.05 | [2.57, 2.75] |
|  | PSEN1-E280A Carrier | 2.80 | 0.04 | [2.72, 2.88] |
|  | PSEN1-E280A Carrier | 2.82 | 0.04 | [2.73, 2.90] |
|  | PSEN1-E280A Carrier MCI | 3.33 | 0.16 | [3.01, 3.65] |
|  | PSEN1-E280A Non-carrier | 2.67 | 0.04 | [2.59, 2.76] |
|  | PSEN1-E280A Carrier MCI | 3.33 | 0.13 | [3.06, 3.59] |
|  | Healthy Elder | 2.88 | 0.08 | [2.72, 3.04] |
|  | Sporadic MCI | 3.11 | 0.08 | [2.96, 3.27] |
| Total Score | PSEN1-E280A Non-carrier | 38.90 | 0.93 | [37.06, 40.74] |
|  | PSEN1-E280A Carrier | 36.96 | 0.85 | [35.28, 38.63] |
|  | PSEN1-E280A Carrier | 36.44 | 0.93 | [34.59, 38.28] |
|  | PSEN1-E280A Carrier MCI | 24.95 | 3.39 | [18.21, 31.68] |
|  | PSEN1-E280A Non-carrier | 38.31 | 0.80 | [36.73, 39.90] |
|  | PSEN1-E280A Carrier MCI | 27.29 | 2.59 | [22.13, 32.46] |
|  | Healthy Elder | 35.29 | 1.80 | [31.64, 38.94] |
|  | Sporadic MCI | 33.86 | 1.73 | [30.35, 37.38] |
| Score 0° condition | PSEN1-E280A Non-carrier | -0.51 | 0.07 | [-0.64, -0.37] |
|  | PSEN1-E280A Carrier | -0.35 | 0.06 | [-0.47, -0.23] |
|  | PSEN1-E280A Carrier | -0.50 | 0.06 | [-0.63, -0.38] |
|  | PSEN1-E280A Carrier MCI | 0.37 | 0.20 | [-0.03, 0.77] |
|  | PSEN1-E280A Non-carrier | -0.50 | 0.06 | [-0.63, -0.38] |
|  | PSEN1-E280A Carrier MCI | 0.37 | 0.20 | [-0.03, 0.77] |
|  | Healthy Elder | -0.41 | 0.15 | [-0.72, -0.10] |
|  | Sporadic MCI | -0.04 | 0.15 | [-0.34, 0.25] |
| Score 90° condition | PSEN1-E280A Non-carrier | 11.63 | 0.57 | [10.50, 12.75] |
|  | PSEN1-E280A Carrier | 10.78 | 0.52 | [9.75, 11.80] |
|  | PSEN1-E280A Carrier | 10.38 | 0.58 | [9.23, 11.53] |
|  | PSEN1-E280A Carrier MCI | 3.90 | 2.11 | [-0.28, 8.09] |
|  | PSEN1-E280A Non-carrier | 11.36 | 0.47 | [10.43, 12.29] |
|  | PSEN1-E280A Carrier MCI | 5.51 | 1.53 | [2.48, 8.55] |
|  | Healthy Elder | 10.09 | 1.08 | [7.91, 12.27] |
|  | Sporadic MCI | 7.83 | 1.04 | [5.73, 9.93] |
| Score 180° Condition | PSEN1-E280A Non-carrier | 1.22 | 0.09 | [1.04, 1.40] |
|  | PSEN1-E280A Carrier | 1.45 | 0.08 | [1.29, 1.61] |
|  | PSEN1-E280A Carrier | 1.48 | 0.09 | [1.31, 1.65] |
|  | PSEN1-E280A Carrier MCI | 2.00 | 0.31 | [1.38, 2.61] |
|  | PSEN1-E280A Non-carrier | 1.28 | 0.08 | [1.11, 1.45] |
|  | PSEN1-E280A Carrier MCI | 1.72 | 0.27 | [1.17, 2.27] |
|  | Healthy Elder | 1.31 | 0.18 | [0.94, 1.67] |
|  | Sporadic MCI | 1.51 | 0.19 | [1.13, 1.90] |
| Span - Forward | PSEN1-E280A Non-carrier | 4.77 | 0.25 | [4.27, 5.27] |
|  | PSEN1-E280A Carrier | 4.32 | 0.23 | [3.87, 4.77] |
|  | PSEN1-E280A Carrier | 4.18 | 0.24 | [3.70, 4.66] |
|  | PSEN1-E280A Carrier MCI | 1.63 | 0.88 | [-0.12, 3.38] |
|  | PSEN1-E280A Non-carrier | 4.75 | 0.25 | [4.26, 5.24] |
|  | PSEN1-E280A Carrier MCI | 2.48 | 0.81 | [0.87, 4.09] |
|  | Healthy Elder | 3.85 | 0.4 | [3.04, 4.66] |
|  | Sporadic MCI | 4.48 | 0.42 | [3.64, 5.32] |
| Span - Backward | PSEN1-E280A Non-carrier | 5.02 | 0.27 | [4.49, 5.55] |
|  | PSEN1-E280A Carrier | 4.43 | 0.24 | [3.95, 4.91] |
|  | PSEN1-E280A Carrier | 4.29 | 0.26 | [3.78, 4.81] |
|  | PSEN1-E280A Carrier MCI | 1.52 | 0.94 | [-0.35, 3.39] |
|  | PSEN1-E280A Non-carrier | 4.98 | 0.25 | [4.48, 5.49] |
|  | PSEN1-E280A Carrier MCI | 2.38 | 0.83 | [0.73, 4.03] |
|  | Healthy Elder | 3.63 | 0.41 | [2.80, 4.47] |
|  | Sporadic MCI | 4.24 | 0.43 | [3.38, 5.11] |
| MRT - Forward | PSEN1-E280A Non-carrier | 8.07 | 0.04 | [7.98, 8.16] |
|  | PSEN1-E280A Carrier | 8.08 | 0.04 | [8.00, 8.16] |
|  | PSEN1-E280A Carrier | 8.07 | 0.03 | [8.01, 8.14] |
|  | PSEN1-E280A Carrier MCI | 8.31 | 0.12 | [8.07, 8.56] |
|  | PSEN1-E280A Non-carrier | 8.07 | 0.05 | [7.97, 8.17] |
|  | PSEN1-E280A Carrier MCI | 8.31 | 0.18 | [7.96, 8.67] |
|  | Healthy Elder | 8.21 | 0.07 | [8.06, 8.35] |
|  | Sporadic MCI | 8.35 | 0.07 | [8.21, 8.50] |
| MRT - Backward | PSEN1-E280A Non-carrier | 8.00 | 0.05 | [7.91, 8.09] |
|  | PSEN1-E280A Carrier | 8.06 | 0.04 | [7.98, 8.14] |
|  | PSEN1-E280A Carrier | 8.07 | 0.04 | [7.98, 8.16] |
|  | PSEN1-E280A Carrier MCI | 7.97 | 0.17 | [7.63, 8.30] |
|  | PSEN1-E280A Non-carrier | 8.00 | 0.04 | [7.92, 8.09] |
|  | PSEN1-E280A Carrier MCI | 8.09 | 0.15 | [7.79, 8.39] |
|  | Healthy Elder | 8.11 | 0.1 | [7.91, 8.30] |
|  | Sporadic MCI | 8.19 | 0.09 | [8.00, 8.38] |

*Note. SE = Standard Error, 95% CI = Confidence Interval at 95%.*
