# Supplementary material for: NavegApp, a serious game for assessing spatial cognition: Diagnostic accuracy in preclinical and prodromal Alzheimer’s disease
Source: PLOS Digit Health. 2026 Jul 10;5(7):e0001521. doi: 10.1371/journal.pdig.0001521 (PMC13354000; doi:10.1371/journal.pdig.0001521)
Supplement: S3 Table — (DOCX) [file pdig.0001521.s003.docx]

## S3 Table. Sensitivity analyses for NavegApp metrics after outlier correction.

Outliers were addressed using a winsorization approach, in which extreme values were replaced by the 5th and 95th percentiles of the respective variable’s distribution. This method reduces the influence of extreme values while maintaining the integrity of the data structure for analysis. The following tables summarize the descriptive statistics of NavegApp’s metrics before and after outlier treatment.

| **Variable** | **Statistics** | **Asymptomatic**  **PSEN1-E280A Carrier** | | **Asymptomatic PSEN1-E280A**  **Non-Carrier** | | **MCI PSEN1–E280A Carriers** | |
| --- | --- | --- | --- | --- | --- | --- | --- |
|  |  | **Original variable** | **After winsorization** | **Original variable** | **After winsorization** | **Original variable** | **After winsorization** |
| Mean Path Length | Min / Max | 31.7 / 78.1 | 18.2 / 101.1 | 30.6 / 77.2 | 26.2 / 92.5 | 16.8 / 93.6 | 8.5 / 98.6 |
|  | Med [IQR] | 50.6 [39.3;64.3] | 50.6 [39.3;64.3] | 46.6 [37.3;58.9] | 46.6 [37.3;58.9] | 63.0 [54.1;78.1] | 63.0 [54.1;78.1] |
|  | Mean (std) | 52.5 (14.5) | 52.8 (15.9) | 49.2 (14.1) | 49.3 (15.0) | 61.3 (25.7) | 60.9 (28.7) |
| Mean Path Time | Min / Max | 1042.2 / 2594.9 | 601.3 / 3376.2 | 1003.7 / 2567.3 | 857.4 / 3084.8 | 553.3 / 3132.5 | 272.7 / 3301.8 |
|  | Med [IQR] | 1675.7 [1301.9;2136.4] | 1675.7 [1301.9;2136.4] | 1542.4 [1234.6;1953.6] | 1542.4 [1234.6;1953.6] | 2100.2 [1821.4;2607.7] | 2100.2 [1821.4;2607.7] |
|  | Mean (std) | 1742.3 (485.7) | 1753.0 (533.2) | 1629.7 (469.9) | 1635.3 (502.4) | 2048.3 (861.6) | 2034.4 (964.3) |
| Mean Error to Goal | Min / Max | 8.6 / 33.5 | 6.9 / 36.6 | 7.3 / 26.2 | 6.2 / 46.4 | 24.2 / 46.0 | 21.1 / 47.0 |
|  | Med [IQR] | 17.6 [11.9;22.7] | 17.6 [11.9;22.7] | 15.0 [11.2;19.1] | 15.0 [11.2;19.1] | 36.5 [33.6;38.7] | 36.5 [33.6;38.7] |
|  | Mean (std) | 18.6 (7.5) | 18.6 (7.8) | 15.4 (5.3) | 15.8 (6.6) | 36.1 (7.0) | 35.9 (8.0) |
| Total Score | Min / Max | 17.0 / 47.0 | 8.0 / 48.0 | 16.8 / 47.2 | 12.0 / 48.0 | 9.8 / 27.5 | 8.0 / 30.0 |
|  | Med [IQR] | 40.0 [30.0;44.0] | 40.0 [30.0;44.0] | 43.0 [33.8;46.0] | 43.0 [33.8;46.0] | 19.5 [16.0;20.8] | 19.5 [16.0;20.8] |
|  | Mean (std) | 36.1 (9.6) | 35.9 (10.0) | 38.6 (9.1) | 38.6 (9.5) | 18.7 (5.6) | 18.8 (6.5) |
| Score 0° Condition | Min / Max | 14.0 / 16.0 | 7.0 / 16.0 | 15.0 / 16.0 | 11.0 / 16.0 | 9.1 / 16.0 | 7.0 / 16.0 |
|  | Med [IQR] | 16.0 [15.0;16.0] | 16.0 [15.0;16.0] | 16.0 [16.0;16.0] | 16.0 [16.0;16.0] | 14.5 [13.8;15.2] | 14.5 [13.8;15.2] |
|  | Mean (std) | 15.7 (0.6) | 15.5 (1.4) | 15.9 (0.4) | 15.8 (0.7) | 14.0 (2.2) | 13.8 (2.9) |
| Score 90° Condition | Min / Max | 0 / 16.0 | 0 / 16.0 | 0 / 16.0 | 0 / 16.0 | 0.4 / 2.0 | 0 / 2.0 |
|  | Med [IQR] | 13.0 [3.5;15.0] | 13.0 [3.5;15.0] | 14.0 [7.8;16.0] | 14.0 [7.8;16.0] | 1.0 [1.0;1.2] | 1.0 [1.0;1.2] |
|  | Mean (std) | 10.2 (6.1) | 10.2 (6.1) | 11.5 (5.4) | 11.5 (5.4) | 1.2 (0.6) | 1.1 (0.6) |
| Score 180° Condition | Min / Max | 1.9 / 15.0 | 0 / 16.0 | 1.0 / 16.0 | 0 / 16.0 | 0 / 10.2 | 0 / 13.0 |
|  | Med [IQR] | 12.0 [6.0;14.0] | 12.0 [6.0;14.0] | 13.0 [10.0;14.0] | 13.0 [10.0;14.0] | 4.0 [0.8;4.2] | 4.0 [0.8;4.2] |
|  | Mean (std) | 10.2 (4.4) | 10.2 (4.6) | 11.3 (4.7) | 11.3 (4.8) | 3.5 (3.4) | 3.9 (4.2) |
| Span - Forward | Min / Max | 0 / 7.0 | 0 / 9.0 | 0 / 8.0 | 0 / 9.0 | 0 / 4.6 | 0 / 5.0 |
|  | Med [IQR] | 5.0 [4.0;5.0] | 5.0 [4.0;5.0] | 5.0 [4.0;6.0] | 5.0 [4.0;6.0] | 0 [0;4.0] | 0 [0;4.0] |
|  | Mean (std) | 4.2 (2.2) | 4.2 (2.3) | 4.7 (2.1) | 4.8 (2.2) | 1.6 (2.2) | 1.6 (2.3) |
| Span - Backward | Min / Max | 0 / 7.0 | 0 / 9.0 | 0 / 8.0 | 0 / 9.0 | 0 / 4.0 | 0 / 4.0 |
|  | Med [IQR] | 5.0 [3.0;6.0] | 5.0 [3.0;6.0] | 6.0 [4.0;6.0] | 6.0 [4.0;6.0] | 0 [0;3.2] | 0 [0;3.2] |
|  | Mean (std) | 4.3 (2.4) | 4.3 (2.4) | 5.0 (2.3) | 5.0 (2.3) | 1.4 (1.9) | 1.4 (1.9) |
| MRT - Forward | Min / Max | 1991.2 / 5229.8 | 1270.0 / 6739.9 | 1571.1 / 6329.1 | 1033.1 / 1.1e+04 | 3138.2 / 4925.5 | 3065.0 / 4980.3 |
|  | Med [IQR] | 3255.9 [2740.2;3878.7] | 3255.9 [2740.2;3878.7] | 3171.2 [2322.9;4089.5] | 3171.2 [2322.9;4089.5] | 3977.4 [3507.9;4772.2] | 3977.4 [3507.9;4772.2] |
|  | Mean (std) | 3387.8 (913.5) | 3407.1 (1026.1) | 3408.4 (1349.2) | 3542.5 (1800.4) | 4085.9 (742.2) | 4083.3 (768.6) |
| MRT - Backward | Min / Max | 1623.6 / 5725.8 | 1292.1 / 9333.6 | 1752.0 / 5340.3 | 1421.5 / 9439.5 | 2094.9 / 6733.2 | 1892.6 / 7710.9 |
|  | Med [IQR] | 3102.7 [2372.4;4408.7] | 3102.7 [2372.4;4408.7] | 2826.5 [2353.8;3598.5] | 2826.5 [2353.8;3598.5] | 2880.3 [2636.9;3882.3] | 2880.3 [2636.9;3882.3] |
|  | Mean (std) | 3383.7 (1230.7) | 3450.4 (1456.7) | 3085.4 (1000.6) | 3180.8 (1376.8) | 3535.2 (1594.0) | 3646.0 (1957.5) |

| **Variable** | **Statistics** | **Healthy Elder** | | **Sporadic MCI** | |
| --- | --- | --- | --- | --- | --- |
|  |  | **Original variable** | **After winsorization** | **Original variable** | **After winsorization** |
| Mean Path Length | Min / Max | 33.4 / 78.2 | 27.4 / 114.1 | 44.8 / 103.6 | 35.8 / 104.2 |
|  | Med [IQR] | 52.1 [45.0;65.8] | 52.1 [45.0;65.8] | 75.8 [58.4;84.1] | 75.8 [58.4;84.1] |
|  | Mean (std) | 54.4 (14.1) | 55.8 (19.0) | 73.1 (17.5) | 72.7 (18.4) |
| Mean Path Time | Min / Max | 1099.3 / 2595.1 | 900.9 / 3806.0 | 1491.4 / 3450.6 | 1181.1 / 3498.0 |
|  | Med [IQR] | 1725.3 [1508.4;2179.9] | 1725.3 [1508.4;2179.9] | 2523.9 [1943.0;2821.4] | 2523.9 [1943.0;2821.4] |
|  | Mean (std) | 1804.4 (470.9) | 1852.6 (635.9) | 2443.8 (587.2) | 2432.4 (620.0) |
| Mean Error to Goal | Min / Max | 9.2 / 39.6 | 6.7 / 43.2 | 15.3 / 40.2 | 14.5 / 40.3 |
|  | Med [IQR] | 17.9 [11.1;27.9] | 17.9 [11.1;27.9] | 23.6 [18.3;27.4] | 23.6 [18.3;27.4] |
|  | Mean (std) | 20.2 (9.7) | 20.2 (10.2) | 24.7 (7.9) | 24.7 (7.9) |
| Total Score | Min / Max | 16.0 / 47.0 | 15.0 / 48.0 | 15.1 / 46.9 | 13.0 / 47.0 |
|  | Med [IQR] | 42.0 [27.0;45.0] | 42.0 [27.0;45.0] | 33.5 [29.0;42.8] | 33.5 [29.0;42.8] |
|  | Mean (std) | 35.4 (11.4) | 35.4 (11.5) | 33.4 (9.7) | 33.3 (10.0) |
| Score 0° Condition | Min / Max | 15.0 / 16.0 | 15.0 / 16.0 | 11.1 / 16.0 | 6.0 / 16.0 |
|  | Med [IQR] | 16.0 [15.0;16.0] | 16.0 [15.0;16.0] | 16.0 [15.0;16.0] | 16.0 [15.0;16.0] |
|  | Mean (std) | 15.7 (0.5) | 15.7 (0.5) | 15.1 (1.5) | 14.9 (2.3) |
| Score 90° Condition | Min / Max | 0 / 16.0 | 0 / 16.0 | 1.0 / 15.0 | 1.0 / 16.0 |
|  | Med [IQR] | 14.0 [3.0;15.0] | 14.0 [3.0;15.0] | 7.5 [1.2;13.5] | 7.5 [1.2;13.5] |
|  | Mean (std) | 10.4 (6.4) | 10.4 (6.4) | 7.4 (5.7) | 7.4 (5.7) |
| Score 180° Condition | Min / Max | 0 / 15.0 | 0 / 16.0 | 0.2 / 15.9 | 0 / 16.0 |
|  | Med [IQR] | 11.0 [5.0;15.0] | 11.0 [5.0;15.0] | 12.0 [10.2;14.0] | 12.0 [10.2;14.0] |
|  | Mean (std) | 9.2 (5.8) | 9.3 (5.9) | 11.1 (4.5) | 11.0 (4.6) |
| Span - Forward | Min / Max | 0 / 7.0 | 0 / 7.0 | 0 / 5.0 | 0 / 6.0 |
|  | Med [IQR] | 5.0 [4.0;6.0] | 5.0 [4.0;6.0] | 4.0 [4.0;5.0] | 4.0 [4.0;5.0] |
|  | Mean (std) | 4.6 (1.9) | 4.6 (1.9) | 3.7 (1.9) | 3.7 (1.9) |
| Span - Backward | Min / Max | 0 / 6.0 | 0 / 6.0 | 0 / 6.0 | 0 / 6.0 |
|  | Med [IQR] | 5.0 [4.0;5.0] | 5.0 [4.0;5.0] | 4.0 [3.0;5.0] | 4.0 [3.0;5.0] |
|  | Mean (std) | 4.3 (1.7) | 4.3 (1.7) | 3.5 (2.1) | 3.5 (2.1) |
| MRT - Forward | Min / Max | 2105.5 / 6697.7 | 1958.8 / 6892.8 | 3164.2 / 6025.0 | 1789.3 / 7471.4 |
|  | Med [IQR] | 3257.7 [2834.8;4581.5] | 3257.7 [2834.8;4581.5] | 4539.0 [3578.7;4863.0] | 4539.0 [3578.7;4863.0] |
|  | Mean (std) | 3798.7 (1420.2) | 3801.0 (1449.6) | 4408.9 (854.0) | 4412.3 (1154.7) |
| MRT - Backward | Min / Max | 2031.0 / 5471.3 | 1361.6 / 5803.5 | 1695.4 / 6712.0 | 1425.7 / 9470.0 |
|  | Med [IQR] | 3545.6 [2553.8;4378.7] | 3545.6 [2553.8;4378.7] | 3829.0 [2631.6;5054.5] | 3829.0 [2631.6;5054.5] |
|  | Mean (std) | 3521.7 (1165.8) | 3505.7 (1245.5) | 3939.1 (1571.3) | 4051.6 (1900.6) |
