# Supplementary material for: NavegApp, a serious game for assessing spatial cognition: Diagnostic accuracy in preclinical and prodromal Alzheimer’s disease
Source: PLOS Digit Health. 2026 Jul 10;5(7):e0001521. doi: 10.1371/journal.pdig.0001521 (PMC13354000; doi:10.1371/journal.pdig.0001521)
Supplement: S4 Table — (DOCX) [file pdig.0001521.s004.docx]

## S4 Table. Spatial cognition performance after outliers correction.

The results of group comparisons for the winsorized variables (i.e., after outlier correction) are presented below. Effect sizes for the observed differences were calculated using Hedges’ g, based on the unstandardized regression coefficients. All linear models were adjusted for covariates, including sex, education, and age.

|  |  | **1. Asymptomatic PSEN1-E280A**  **non-carriers**  **(n = 76)** | **2. Asymptomatic  PSEN1-E280A carriers**  **(n = 99)** | **3. MCI**  **PSEN1-E280A carriers**  **(n = 8)** | **1 Vs 2** | **1 Vs 3** | **2 vs 3** |
| --- | --- | --- | --- | --- | --- | --- | --- |
|  |  | **Med [IQR]** | **Med [IQR]** | **Med [IQR]** | **Hedges' g [CI_95%_]** | **Hedges' g [CI_95%_]** | **Hedges' g [CI_95%_]** |
| **Gamified Hidden Goal Task (gHGT)** | | |  |  |  |  |  |
|  | Mean Path Distance | 46.6  [37.3, 58.9] | 50.6  [39.3, 64.3] | 63.0  [54.1, 78.1] | 0.22  [-0.08, 0.52] | -0.01  [-0.73, 0.71] | -0.01  [-0.73, 0.71] |
|  | Mean Path Time | 1542.4  [1234.6, 1953.6] | 1675.7  [1301.9, 2136.4] | 2100.2  [1821.4, 2607.7] | 0.22  [-0.07, 0.52] | 0.39  [-0.34, 1.12] | 0.01  [-0.71, 0.73] |
|  | Mean Error to Goal | 15.0  [11.2, 19.1] | 17.6  [11.9, 22.7] | 36.5  [33.6, 38.7] | 0.38  [0.08, 0.69] | 1.83  [1.05, 2.61] | 1.27  [0.53, 2.01] |
| **Gamified Mental Rotation Task (gMRT)** | | |  |  |  |  |  |
|  | Total Score | 43.0  [33.8, 46.0] | 40.0  [30.0, 44.0] | 19.5  [16.0, 20.8] | -0.2  [-0.5, 0.1] | -1.12  [-1.87, -0.37] | -1.23  [-1.97, -0.49] |
|  | Score 0° Condition | 16.0  [16.0, 16.0] | 16.0  [15.0, 16.0] | 14.5  [13.8, 15.2] | 0.3  [0.0, 0.6] | 1.76  [1.00, 2.52] | 1.17  [0.42, 1.92] |
|  | Score 90° Condition | 14.0 [7.8, 16.0] | 13.0 [3.5, 15.0] | 1.0 [1.0, 1.2] | -0.15  [-0.45, 0.15] | -1.00  [-1.75, -0.26] | -1.04  [-1.78, -0.31] |
|  | Score 180° Condition | 13.0  [10.0, 14.0] | 12.0  [6.0, 14.0] | 4.0  [0.8, 4.2] | 0.33  [0.02, 0.63] | 0.56  [-0.18, 1.29] | 0.85  [0.12, 1.58] |
| **Gamified Corsi Task (gCorsi)** | |  |  |  |  |  |  |
|  | Span - Forward | 5.0 [4.0, 6.0] | 5.0 [4.0, 5.0] | 0.0 [0.0, 4.0] | -0.23  [-0.53, 0.07] | -1.02  [-1.77, -0.28] | -1.19  [-1.92, -0.45] |
|  | Span - Backward | 6.0 [4.0, 6.0] | 5.0 [3.0, 6.0] | 0.0 [0.0, 3.2] | -0.26  [-0.56, 0.04] | -1.1  [-1.85, -0.36] | -1.18  [-1.92, -0.44] |
|  | MRT - Forward | 3171.2  [2322.9, 4089.5] | 3255.9  [2740.2, 3878.7] | 3977.4  [3507.9, 4772.2] | 0.07  [-0.23, 0.37] | 0.64  [-0.09, 1.38] | 0.85  [0.12, 1.58] |
|  | MRT - Backward | 2826.5  [2353.8, 3598.5] | 3102.7  [2372.4, 4408.7] | 2880.3  [2636.9, 3882.3] | 0.19  [-0.11, 0.49] | 0.24  [-0.49, 0.97] | -0.24 [-0.97, 0.48] |

*Note. Med [IQR] = Median [Interquartile Range]; CI_95%_ = Confidence interval at 95%.*
