# Supplementary material for: NavegApp, a serious game for assessing spatial cognition: Diagnostic accuracy in preclinical and prodromal Alzheimer’s disease
Source: PLOS Digit Health. 2026 Jul 10;5(7):e0001521. doi: 10.1371/journal.pdig.0001521 (PMC13354000; doi:10.1371/journal.pdig.0001521)
Supplement: S6 Table — (DOCX) [file pdig.0001521.s006.docx]

## S6 Table. Diagnostic accuracy by genetic status.

The following section presents a comparison of group performance on NavegApp. Results for asymptomatic participants are disaggregated by genetic status to provide detailed insights into their performance metrics.

|  |  | **PSEN1-E280A Carriers Vs.  PSEN1-E280A non-carriers** | **PSEN1-E280A carriers Vs.  MCI PSEN1-E280A carriers** | **PSEN1-E280A carriers Vs.  MCI PSEN1-E280A carriers** | **Healthy Elder Vs.  Sporadic MCI** |
| --- | --- | --- | --- | --- | --- |
|  |  |  |  |  |  |
| **Gamified Hidden Goal Task (gHGT)** | | | | | |
|  | Mean Path Distance | 0.57 [0.48, 0.65] | 0.63 [0.36, 0.91] | 0.68 [0.40, 0.96] | 0.77 [0.62, 0.91] |
|  | Mean Path Time | 0.57 [0.48, 0.65] | 0.64 [0.37, 0.91] | 0.69 [0.41, 0.97] | 0.77 [0.62, 0.92] |
|  | Mean Error to Goal | 0.60 [0.52, 0.69] | 0.94 [0.85, 1.00] | 0.97 [0.92, 1.00] | 0.65 [0.47, 0.85] |
| **Gamified Mental Rotation Task (gMRT)** | | | | | |
|  | Total Score | 0.59 [0.51, 0.68] | 0.91 [0.84, 0.91] | 0.93 [0.87, 0.99] | 0.56 [0.38, 0.75] |
|  | Score 0° Condition | 0.56 [0.50, 0.62] | 0.78 [0.60, 0.97] | 0.83 [0.65, 1.00] | 0.59 [0.44, 0.73] |
|  | Score 90° Condition | 0.56 [0.48, 0.65] | 0.86 [0.79, 0.92] | 0.91 [0.86, 0.97] | 0.64 [0.46, 0.81] |
|  | Score 180° Condition | 0.59 [0.51, 0.68] | 0.84 [0.69, 0.98] | 0.86 [0.75, 0.97] | 0.45 [0.27, 0.63] |
| **Gamified Corsi Task (gCorsi)** | | | | | |
|  | Span - Forward | 0.57 [0.49, 0.65] | 0.80 [0.68, 0.93] | 0.85 [0.73, 0.96] | 0.63 [0.47, 0.80] |
|  | Span - Backward | 0.59 [0.50, 0.67] | 0.83 [0.73, 0.92] | 0.89 [0.81, 0.96] | 0.62 [0.45, 0.79] |
|  | RT - Forward | 0.52 [0.43, 0.61] | 0.71 [0.56, 0.87] | 0.70 [0.56, 0.84] | 0.67 [0.50, 0.84] |
|  | RT - Backward | 0.56 [0.47, 0.64] | 0.51 [0.28, 0.73] | 0.56 [0.32, 0.79] | 0.56 [0.40, 0.74] |

*Note. RT = Reaction Time, MCI = Mild Cognitive Impairment*
