# Supplementary material for: NavegApp, a serious game for assessing spatial cognition: Diagnostic accuracy in preclinical and prodromal Alzheimer’s disease
Source: PLOS Digit Health. 2026 Jul 10;5(7):e0001521. doi: 10.1371/journal.pdig.0001521 (PMC13354000; doi:10.1371/journal.pdig.0001521)
Supplement: S7 Table — (DOCX) [file pdig.0001521.s007.docx]

## S7 Table. Complementary diagnostic accuracy metrics.

In addition to the AUC-ROC, supplementary diagnostic accuracy measures were calculated for each spatial cognition (SC) metric obtained from NavegApp. The optimal cutoff points were determined using the Youden Index. These findings should be considered preliminary and require validation in future research, as NavegApp remains under development.

|  |  | **AUC-ROC (IC95%)** | **Se (IC95%)** | **Sp (IC95%)** | **PPV (IC95%)** | **NPV (IC95%)** |
| --- | --- | --- | --- | --- | --- | --- |
| **PSEN1-E280A Carriers Vs PSEN1-E280A Non-carriers** | | | | | | |
|  | Mean Path Distance | 0.57 (0.48, 0.65) | 0.83 (0.73, 0.91) | 0.30 (0.21, 0.40) | 0.48 (0.39, 0.57) | 0.70 (0.54, 0.83) |
|  | Mean Path Time | 0.57 (0.48, 0.65) | 0.83 (0.73, 0.91) | 0.30 (0.21, 0.40) | 0.48 (0.39, 0.57) | 0.70 (0.54, 0.83) |
|  | Mean Error to Goal | 0.6 (0.52, 0.69) | 0.78 (0.67, 0.86) | 0.42 (0.33, 0.53) | 0.51 (0.41, 0.60) | 0.71 (0.58, 0.82) |
|  | Total Score | 0.59 (0.51, 0.68) | 0.61 (0.49, 0.72) | 0.55 (0.44, 0.65) | 0.51 (0.40, 0.61) | 0.64 (0.53, 0.74) |
|  | Score 0° Condition | 0.56 (0.5, 0.62) | 0.96 (0.89, 0.99) | 0.07 (0.03, 0.14) | 0.44 (0.37, 0.52) | 0.70 (0.35, 0.93) |
|  | Score 90° Condition | 0.56 (0.48, 0.65) | 0.88 (0.79, 0.94) | 0.21 (0.14, 0.31) | 0.46 (0.38, 0.55) | 0.70 (0.51, 0.85) |
|  | Score 180° Condition | 0.59 (0.51, 0.68) | 0.70 (0.58, 0.80) | 0.42 (0.33, 0.53) | 0.48 (0.39, 0.58) | 0.65 (0.52, 0.76) |
|  | Span - Forward | 0.57 (0.49, 0.65) | 0.71 (0.60, 0.81) | 0.33 (0.24, 0.44) | 0.45 (0.36, 0.54) | 0.60 (0.46, 0.73) |
|  | Span - Backward | 0.59 (0.5, 0.67) | 0.83 (0.73, 0.91) | 0.28 (0.20, 0.38) | 0.47 (0.38, 0.56) | 0.68 (0.52, 0.82) |
|  | MRT - Forward | 0.52 (0.43, 0.61) | 0.42 (0.31, 0.54) | 0.73 (0.64, 0.82) | 0.55 (0.42, 0.68) | 0.62 (0.53, 0.71) |
|  | MRT - Backward | 0.56 (0.47, 0.64) | 0.82 (0.72, 0.90) | 0.33 (0.24, 0.44) | 0.49 (0.40, 0.58) | 0.71 (0.56, 0.84) |
| **PSEN1-E280A Carriers Vs MCI PSEN1-E280A Carriers** | | | | | | |
|  | Mean Path Distance | 0.63 [0.36, 0.91] | 0.69 (0.59, 0.78) | 0.75 (0.35, 0.97) | 0.97 (0.90, 1.00) | 0.16 (0.06, 0.32) |
|  | Mean Path Time | 0.64 [0.37, 0.91] | 0.70 (0.60, 0.79) | 0.62 (0.24, 0.91) | 0.96 (0.88, 0.99) | 0.14 (0.05, 0.30) |
|  | Mean Error to Goal | 0.94 [0.85, 1.00] | 0.89 (0.81, 0.94) | 0.88 (0.47, 1.00) | 0.99 (0.94, 1.00) | 0.39 (0.17, 0.64) |
|  | Total Score | 0.91 [0.84, 0.91] | 0.76 (0.66, 0.84) | 0.88 (0.47, 1.00) | 0.99 (0.93, 1.00) | 0.23 (0.10, 0.41) |
|  | Score 0° Condition | 0.78 [0.6, 0.97] | 0.93 (0.86, 0.97) | 0.50 (0.16, 0.84) | 0.96 (0.90, 0.99) | 0.36 (0.11, 0.69) |
|  | Score 90° Condition | 0.86 [0.79, 0.92] | 0.83 (0.74, 0.90) | 0.75 (0.35, 0.97) | 0.98 (0.92, 1.00) | 0.26 (0.10, 0.48) |
|  | Score 180° Condition | 0.84 [0.69, 0.98] | 0.84 (0.75, 0.90) | 0.75 (0.35, 0.97) | 0.98 (0.92, 1.00) | 0.27 (0.11, 0.50) |
|  | Span - Forward | 0.8 [0.68, 0.93] | 0.78 (0.68, 0.86) | 0.62 (0.24, 0.91) | 0.96 (0.89, 0.99) | 0.19 (0.06, 0.38) |
|  | Span - Backward | 0.83 [0.73, 0.92] | 0.72 (0.62, 0.80) | 0.75 (0.35, 0.97) | 0.97 (0.90, 1.00) | 0.18 (0.07, 0.35) |
|  | MRT - Forward | 0.71 [0.56, 0.87] | 0.47 (0.37, 0.57) | 0.86 (0.42, 1.00) | 0.98 (0.89, 1.00) | 0.10 (0.04, 0.21) |
|  | MRT - Backward | 0.51 [0.28, 0.73] | 0.34 (0.25, 0.45) | 0.86 (0.42, 1.00) | 0.97 (0.85, 1.00) | 0.09 (0.03, 0.18) |
| **PSEN1-E280A Non-carriers Vs MCI PSEN1-E280A Carriers** | | | | | | |
|  | Mean Path Distance | 0.68 [0.4, 0.96] | 0.79 (0.68, 0.87) | 0.75 (0.35, 0.97) | 0.97 (0.89, 1.00) | 0.27 (0.11, 0.50) |
|  | Mean Path Time | 0.69 [0.41, 0.97] | 0.83 (0.73, 0.91) | 0.62 (0.24, 0.91) | 0.95 (0.87, 0.99) | 0.28 (0.10, 0.53) |
|  | Mean Error to Goal | 0.97 [0.92, 1] | 0.97 (0.91, 1.00) | 0.88 (0.47, 1.00) | 0.99 (0.93, 1.00) | 0.78 (0.40, 0.97) |
|  | Total Score | 0.93 [0.87, 0.99] | 0.82 (0.71, 0.90) | 0.88 (0.47, 1.00) | 0.98 (0.91, 1.00) | 0.33 (0.15, 0.57) |
|  | Score 0° Condition | 0.83 [0.65, 1] | 0.96 (0.89, 0.99) | 0.50 (0.16, 0.84) | 0.95 (0.87, 0.99) | 0.57 (0.18, 0.90) |
|  | Score 90° Condition | 0.91 [0.86, 0.97] | 0.89 (0.80, 0.95) | 0.75 (0.35, 0.97) | 0.97 (0.90, 1.00) | 0.43 (0.18, 0.71) |
|  | Score 180° Condition | 0.86 [0.75, 0.97] | 0.86 (0.76, 0.93) | 0.75 (0.35, 0.97) | 0.97 (0.90, 1.00) | 0.35 (0.14, 0.62) |
|  | Span - Forward | 0.85 [0.73, 0.96] | 0.84 (0.74, 0.92) | 0.62 (0.24, 0.91) | 0.96 (0.87, 0.99) | 0.29 (0.10, 0.56) |
|  | Span - Backward | 0.89 [0.81, 0.96] | 0.83 (0.73, 0.91) | 0.75 (0.35, 0.97) | 0.97 (0.89, 1.00) | 0.32 (0.13, 0.57) |
|  | MRT - Forward | 0.7 [0.56, 0.84] | 0.47 (0.36, 0.59) | 1.00 (0.63, 1.00) | 1.00 (0.90, 1.00) | 0.17 (0.07, 0.30) |
|  | MRT - Backward | 0.56 [0.32, 0.79] | 0.33 (0.23, 0.45) | 0.88 (0.47, 1.00) | 0.96 (0.80, 1.00) | 0.12 (0.05, 0.23) |
| **Healthy Elders Vs Sporadic MCI** | | | | | | |
|  | Mean Path Distance | 0.77 [0.62, 0.91] | 0.90 (0.70, 0.99) | 0.55 (0.32, 0.76) | 0.66 (0.46, 0.82) | 0.86 (0.57, 0.98) |
|  | Mean Path Time | 0.77 [0.62, 0.92] | 0.90 (0.70, 0.99) | 0.59 (0.36, 0.79) | 0.68 (0.48, 0.84) | 0.87 (0.60, 0.98) |
|  | Mean Error to Goal | 0.65 [0.47, 0.85] | 0.43 (0.22, 0.66) | 0.91 (0.71, 0.99) | 0.82 (0.48, 0.98) | 0.62 (0.44, 0.79) |
|  | Total Score | 0.56 [0.38, 0.75] | 0.48 (0.26, 0.70) | 0.73 (0.50, 0.89) | 0.62 (0.35, 0.85) | 0.59 (0.39, 0.78) |
|  | Score 0° Condition | 0.59 [0.44, 0.73] | 1.00 (0.84, 1.00) | 0.14 (0.03, 0.35) | 0.52 (0.36, 0.68) | 1.00 (0.29, 1.00) |
|  | Score 90° Condition | 0.64 [0.46, 0.81] | 0.67 (0.43, 0.85) | 0.64 (0.41, 0.83) | 0.64 (0.41, 0.83) | 0.67 (0.43, 0.85) |
|  | Score 180° Condition | 0.45 [0.27, 0.63] | 0.48 (0.26, 0.70) | 0.41 (0.21, 0.64) | 0.43 (0.23, 0.66) | 0.45 (0.23, 0.68) |
|  | Span - Forward | 0.63 [0.47, 0.8] | 0.62 (0.38, 0.82) | 0.55 (0.32, 0.76) | 0.57 (0.34, 0.77) | 0.60 (0.36, 0.81) |
|  | Span - Backward | 0.62 [0.45, 0.79] | 0.90 (0.70, 0.99) | 0.23 (0.08, 0.45) | 0.53 (0.35, 0.70) | 0.71 (0.29, 0.96) |
|  | MRT - Forward | 0.67 [0.5, 0.84] | 0.52 (0.30, 0.74) | 0.90 (0.70, 0.99) | 0.85 (0.55, 0.98) | 0.66 (0.46, 0.82) |
|  | MRT - Backward | 0.56 [0.39, 0.74] | 0.86 (0.64, 0.97) | 0.32 (0.14, 0.55) | 0.55 (0.36, 0.72) | 0.70 (0.35, 0.93) |
