# Supplementary material for: NavegApp, a serious game for assessing spatial cognition: Diagnostic accuracy in preclinical and prodromal Alzheimer’s disease
Source: PLOS Digit Health. 2026 Jul 10;5(7):e0001521. doi: 10.1371/journal.pdig.0001521 (PMC13354000; doi:10.1371/journal.pdig.0001521)
Supplement: S8 Table — (DOCX) [file pdig.0001521.s008.docx]

**S8 Table.** Adjusted confidence intervals for group comparisons.

This section presents the adjusted confidence intervals for the Hedges’ g effect-size comparisons. Adjusted intervals were computed as a conservative sensitivity analysis to account for multiple testing across NavegApp-derived metrics and between-group contrasts, including comparisons among asymptomatic PSEN1-E280A non-carriers, asymptomatic PSEN1-E280A carriers, PSEN1-E280A carriers with MCI, cognitively healthy older adults, and participants with sporadic MCI.

|  |  | **PSEN1-E280A Carriers Vs. PSEN1-E280A non-carriers** | **PSEN1-E280A carriers Vs. MCI PSEN1-E280A carriers** | **PSEN1-E280A carriers Vs. MCI PSEN1-E280A carriers** | **Healthy Elder Vs.  Sporadic MCI** |
| --- | --- | --- | --- | --- | --- |
|  |  |  |  |  |  |
|  |  | **Hedges' g [IC_99%_]** | **Hedges' g [IC_99%_]** | **Hedges' g [IC_99%_]** | **Hedges' g [IC_99%_]** |
| **Gamified Hidden Goal Task (gHGT)** | | | | | |
|  | Mean Path Distance | 0.21 [-0.28;0.71] | 0.31 [-0.90;1.52] | -0.16 [-1.36;1.04] | 0.95 [-0.10;2.00] |
|  | Mean Path Time | 0.21 [-0.28;0.71] | 0.33 [-0.75;1.41] | -0.14 [-1.34;1.06] | 0.97 [-0.08;2.02] |
|  | Mean Error to Goal | 0.33 [-0.17;0.83] | 1.52 [0.25;2.79] | 1.14 [-0.08;2.36] | 0.53 [-0.48;1.54] |
| **Gamified Mental Rotation Task (gMRT)** | | |  |  |  |
|  | Total Score | -0.20 [-0.69;0.30] | -1.04 [-2.28;0.19] | -1.10 [-2.32;0.12] | -0.13 [-1.12;0.86] |
|  | Score 0° Condition | 0.25 [-0.25;0.75] | 1.46 [0.22;2.70] | 0.78 [-0.44;2.01] | 0.49 [-0.52;1.49] |
|  | Score 90° Condition | -0.15 [-0.64;0.35] | -1.01 [-2.24;0.23] | -1.05 [-2.27;0.17] | -0.36 [-1.36;0.64] |
|  | Score 180° Condition | 0.26 [-0.24;0.76] | 0.46 [-0.75;1.68] | 0.59 [-0.61;1.79] | -0.20 [-1.20;0.79] |
| **Gamified Corsi Task (gCorsi)** | | | | | |
|  | Span - Forward | -0.20 [-0.70;0.30] | -0.99 [-2.23;0.24] | -1.11 [-2.33;0.11] | -0.32 [-1.32;0.68] |
|  | Span - Backward | -0.25 [-0.75;0.25] | -1.08 [-2.32;0.16] | -1.14 [-2.37;0.08] | -0.31 [-1.31;0.69] |
|  | MRT - Forward | 0.02 [-0.47;0.52] | 0.55 [-0.67;1.77] | 0.82 [-0.39;2.03] | 0.43 [-0.58;1.43] |
|  | MRT - Backward | 0.15 [-0.35;0.64] | 0.22 [-0.99;1.43] | -0.25 [-1.44;0.95] | 0.19 [-0.80;1.18] |
