# Supplementary material for: NavegApp, a serious game for assessing spatial cognition: Diagnostic accuracy in preclinical and prodromal Alzheimer’s disease
Source: PLOS Digit Health. 2026 Jul 10;5(7):e0001521. doi: 10.1371/journal.pdig.0001521 (PMC13354000; doi:10.1371/journal.pdig.0001521)
Supplement: S1 Fig — (DOCX) [file pdig.0001521.s009.docx]

## S1 Fig. Missing data management.

During the initial statistical exploratory analysis, missing data were identified in the mean reaction time for both the forward and backward conditions of the gCorsi task. As the proportion of missing data was below 5%, simple imputation methods using the mean and median were applied. The results of these imputations are presented below. No notable differences were observed in the distribution of the variables or their descriptive statistics following imputation.

**
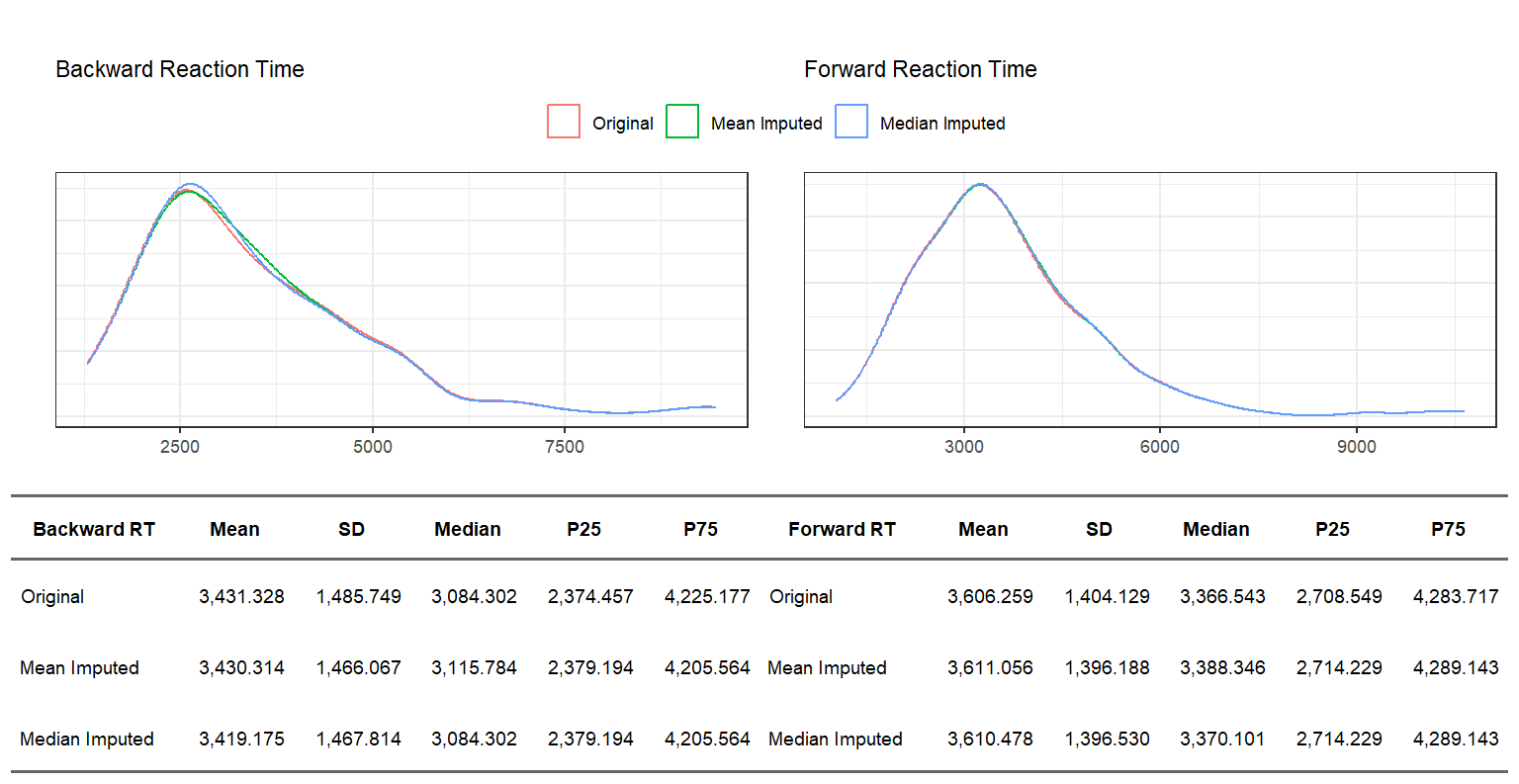
**
