# Supplementary figures and images for: NavegApp, a serious game for assessing spatial cognition: Diagnostic accuracy in preclinical and prodromal Alzheimer’s disease
Source: PLOS Digit Health. 2026 Jul 10;5(7):e0001521. doi: 10.1371/journal.pdig.0001521 (PMC13354000; doi:10.1371/journal.pdig.0001521)

## S2 Fig. Linear mixed model residuals for the Path Length - PSEN1-E280A Cohort.

**
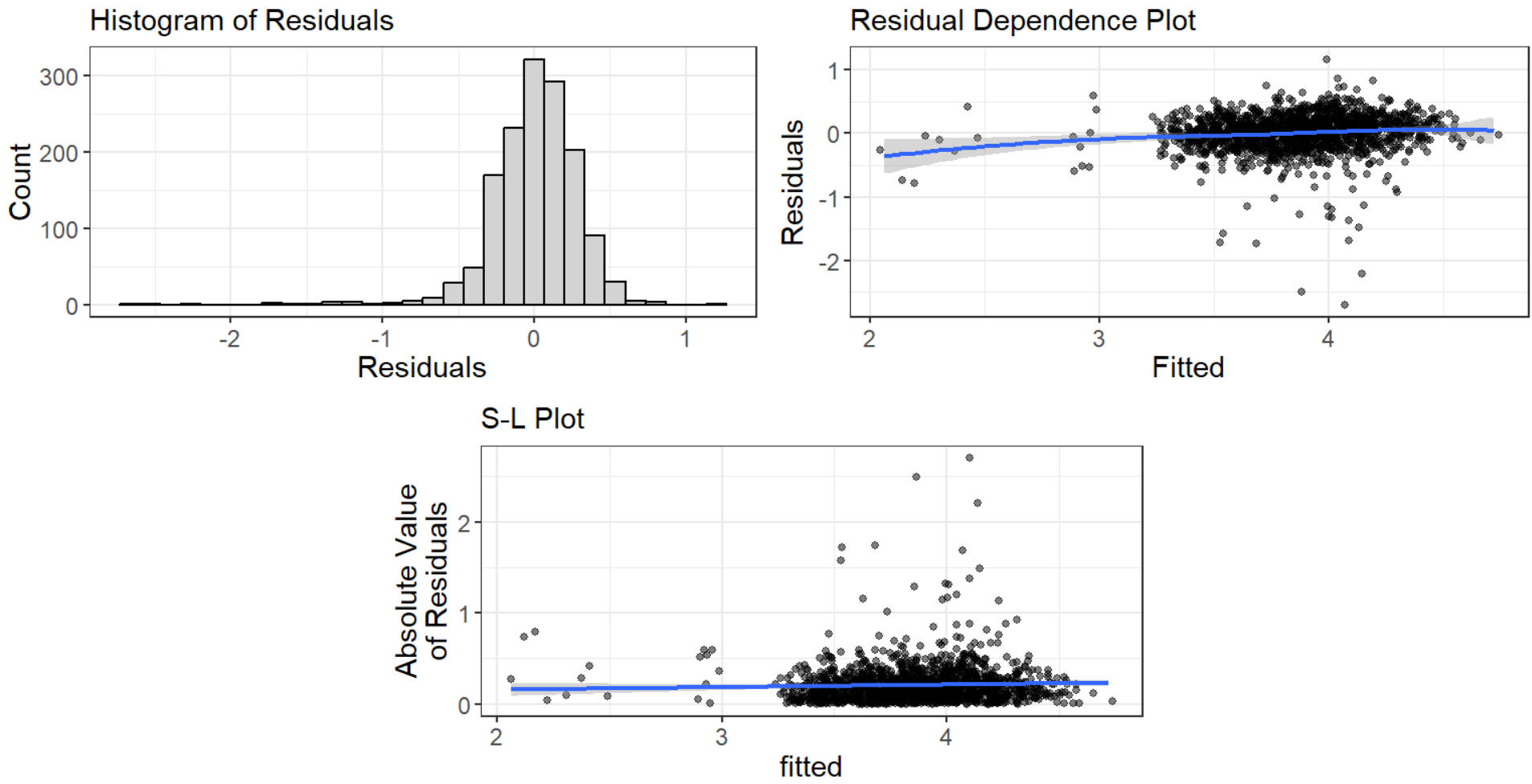
**

Supplement: S2 Fig — (DOCX) [file pdig.0001521.s010.docx]

## S3 Fig. Linear mixed model residuals for the Path Length - Healthy elder and Sporadic MCI.

**
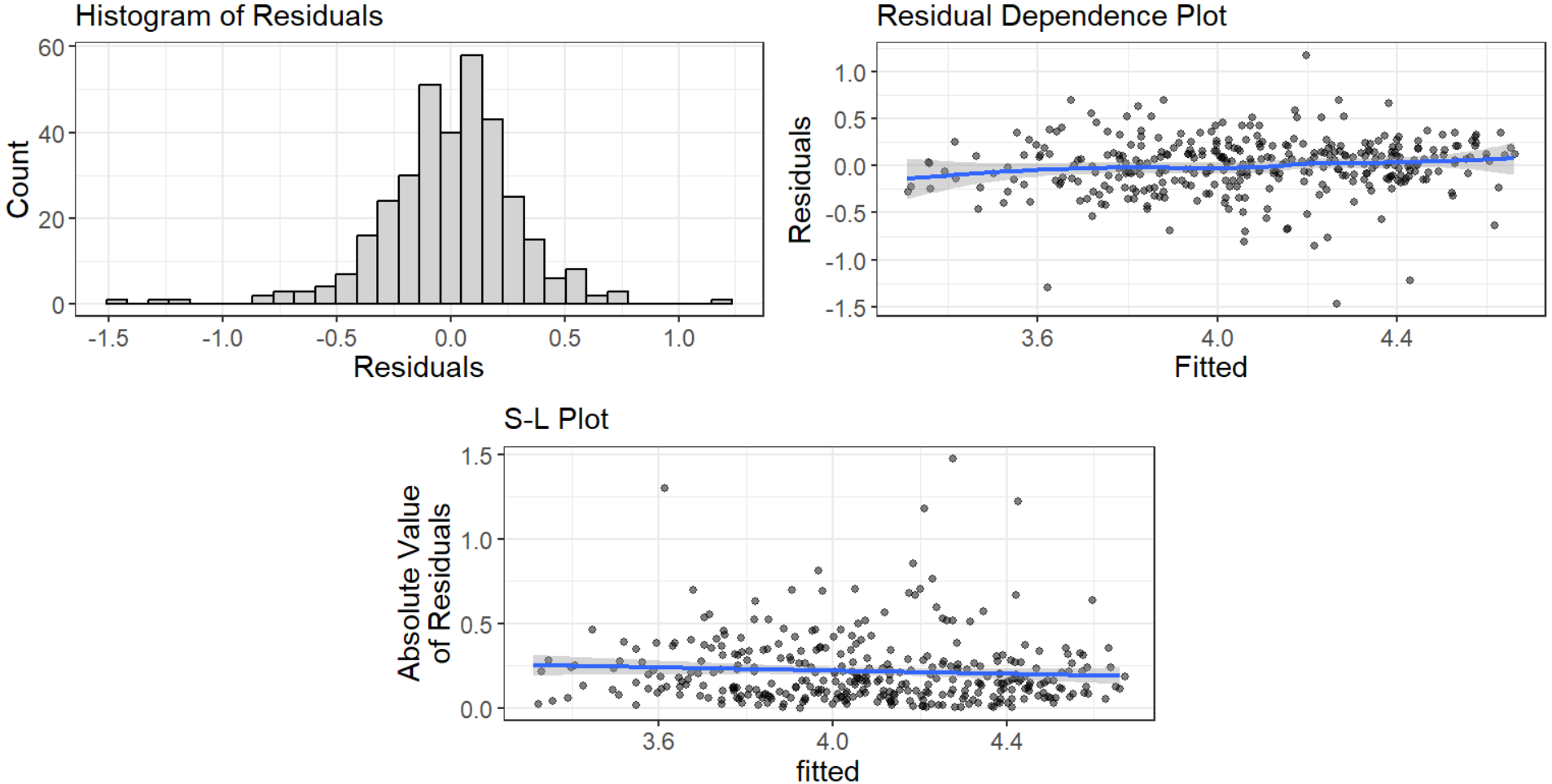
**

Supplement: S3 Fig — (DOCX) [file pdig.0001521.s011.docx]

## S4 Fig. Linear mixed model residuals for the Path Time - PSEN1-E280A Cohort.

**
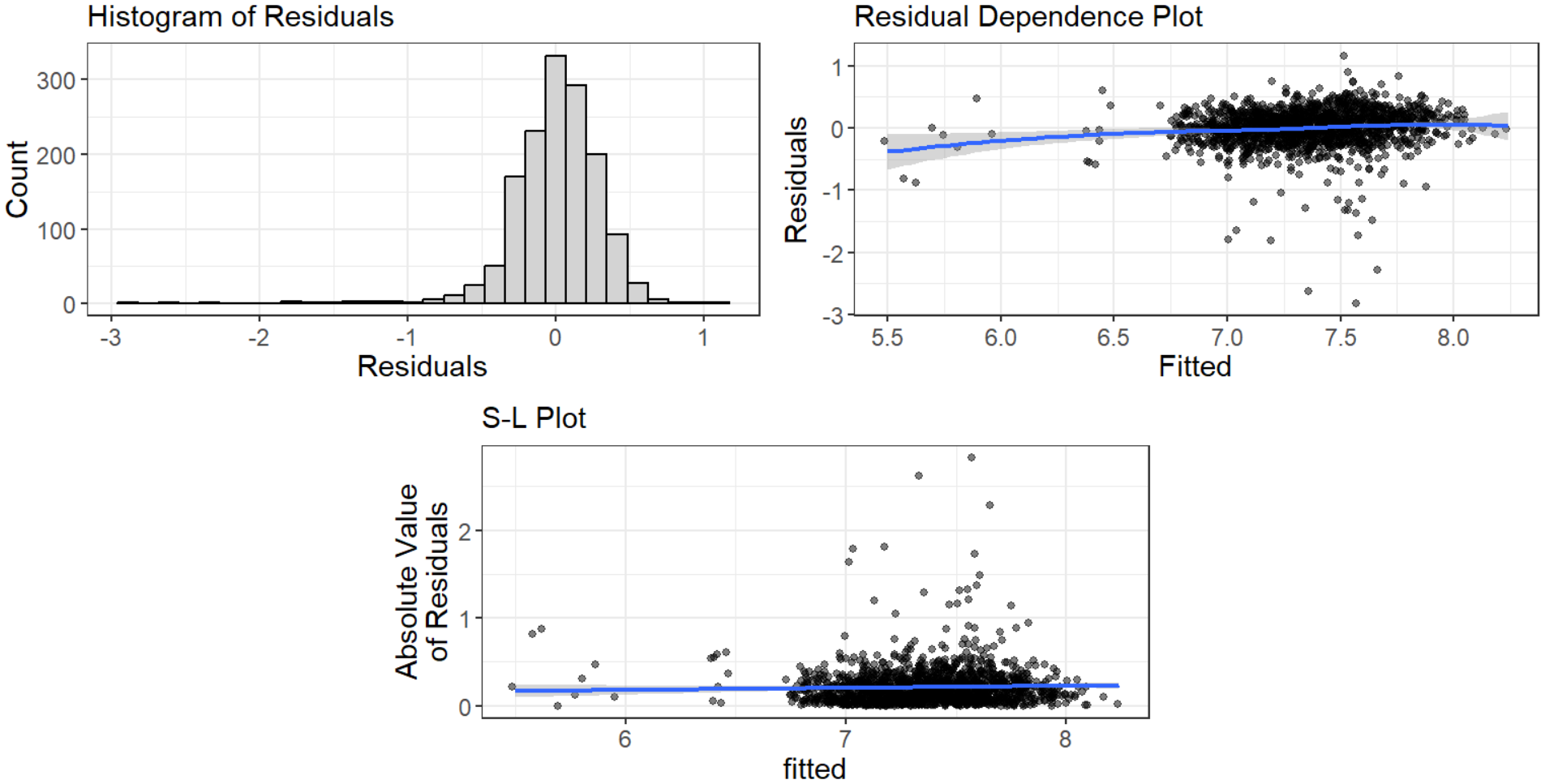
**

Supplement: S4 Fig — (DOCX) [file pdig.0001521.s012.docx]

## S5 Fig. Linear mixed model residuals for the Path Time - Healthy elder and Sporadic MCI.

**
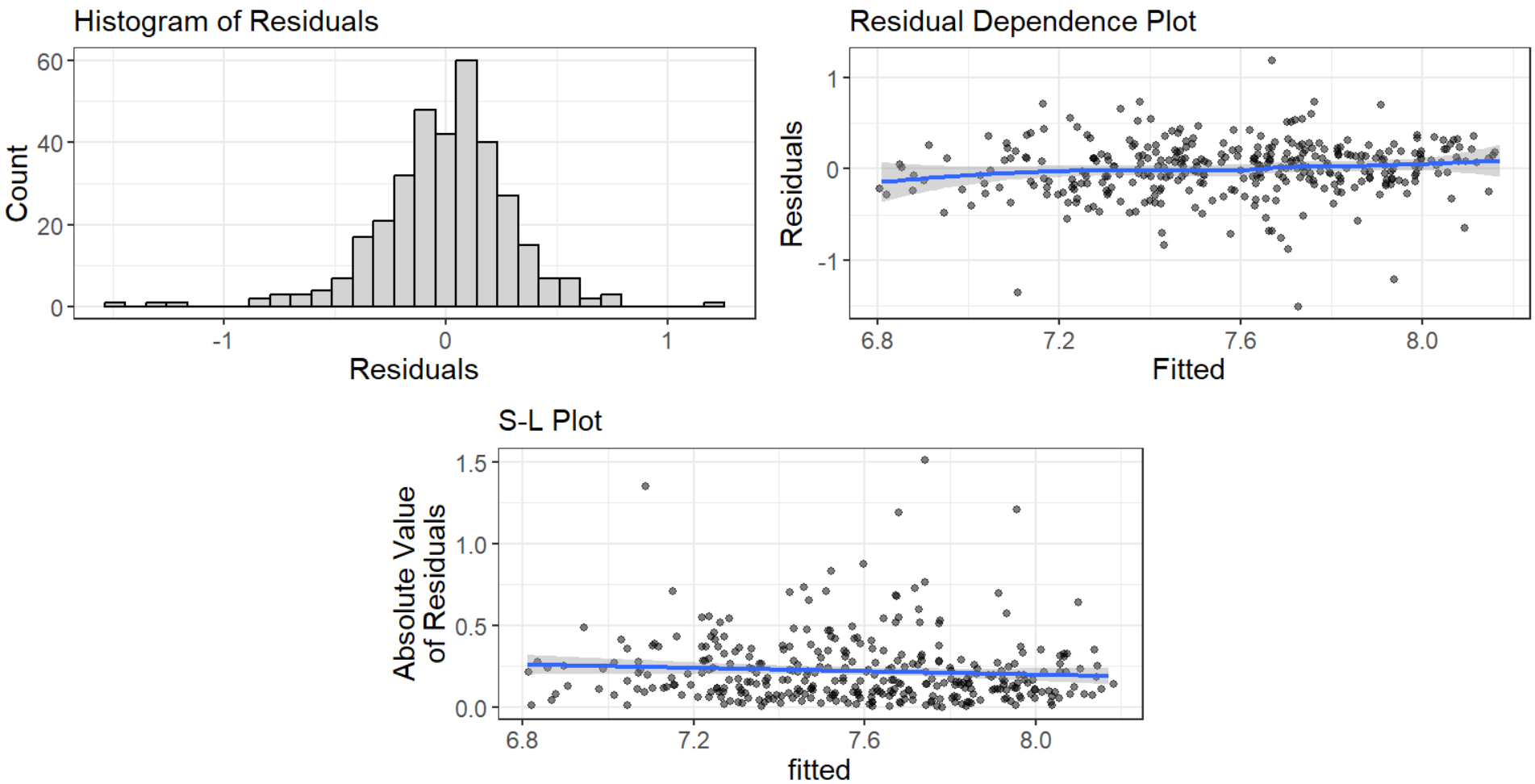
**

Supplement: S5 Fig — (DOCX) [file pdig.0001521.s013.docx]

**S6 Fig.** Linear mixed model residuals for the mean Error to the goal - PSEN1-E280A Cohort.

**
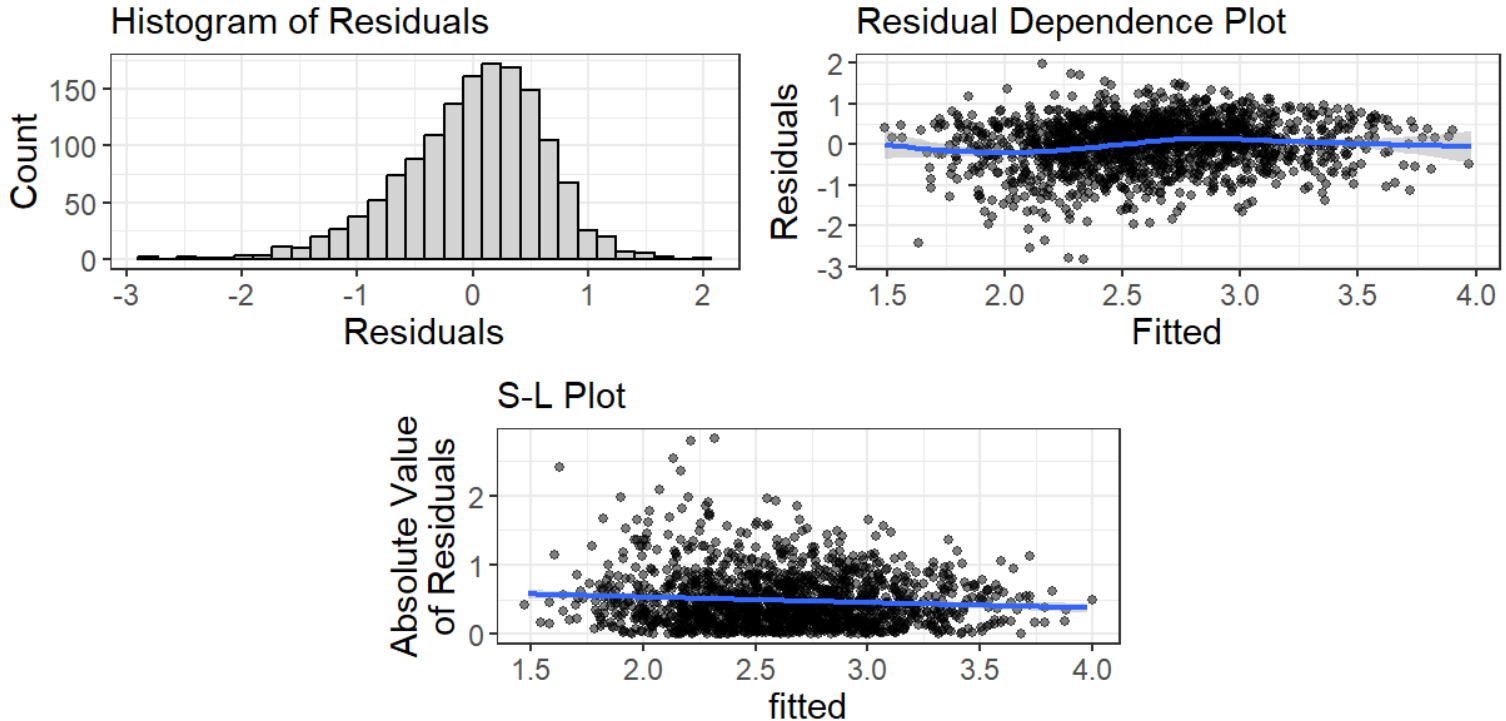
**

Supplement: S6 Fig — (DOCX) [file pdig.0001521.s014.docx]

**S7 Fig.** Linear mixed model residuals for the mean Error to the goal - Healthy elder and Sporadic MCI.

**
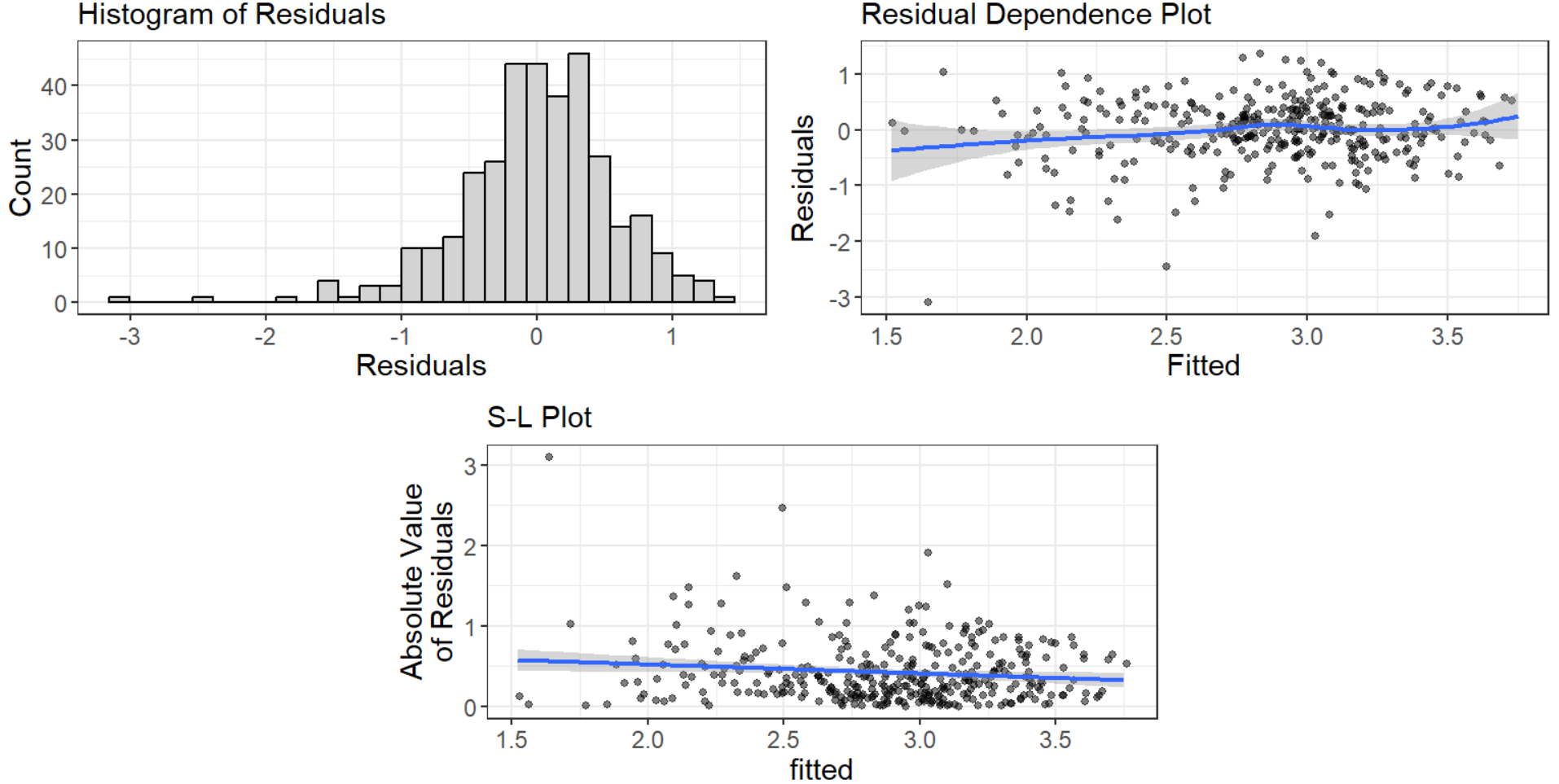
**

Supplement: S7 Fig — (DOCX) [file pdig.0001521.s015.docx]
